# Thermodynamics and Kinetics of Ion Permeation in Wild-Type and Mutated Open Active Conformation of the Human α7 Nicotinic Receptor

**DOI:** 10.1021/acs.jcim.0c00549

**Published:** 2020-08-17

**Authors:** Grazia Cottone, Letizia Chiodo, Luca Maragliano

**Affiliations:** † Department of Physics and Chemistry-Emilio Segrè, University of Palermo, Viale delle Scienze Ed. 17, 90128 Palermo, Italy; ‡ Department of Engineering, Campus Bio-Medico University of Rome, Via Á. del Portillo 21, 00128 Rome, Italy; ¶ Center for Synaptic Neuroscience and Technology (NSYN@UniGe), Istituto Italiano di Tecnologia, Largo Rosanna Benzi, 10, 16132 Genova, Italy; ∥ IRCCS Ospedale Policlinico San Martino, Largo Rosanna Benzi, 10, 16132 Genova, Italy

## Abstract

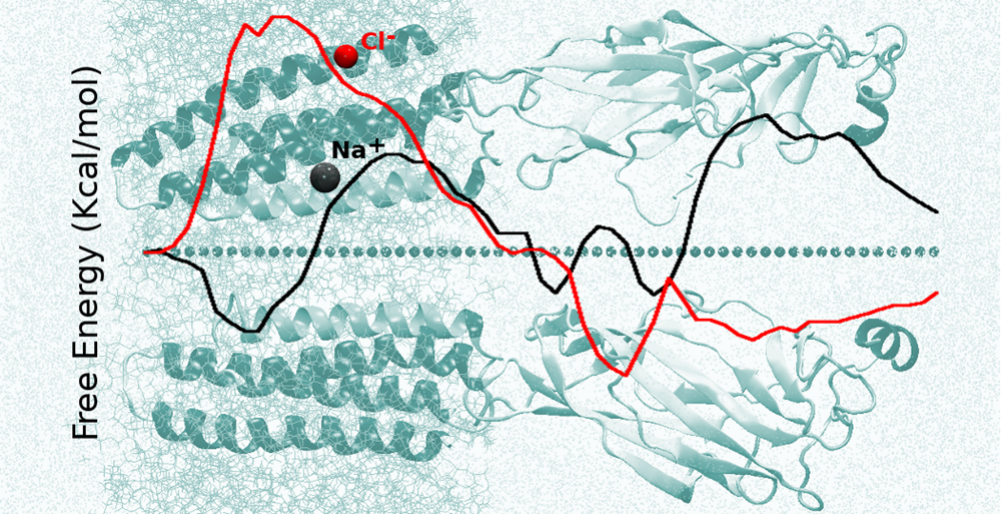

Molecular studies of human pentameric ligand-gated ion channels (LGICs) expressed in neurons and at neuromuscular junctions are of utmost importance in the development of therapeutic strategies for neurological disorders. We focus here on the nicotinic acetylcholine receptor nAChR-α7, a homopentameric channel widely expressed in the human brain, with a proven role in a wide spectrum of disorders including schizophrenia and Alzheimer’s disease. By exploiting an all-atom structural model of the full (transmembrane and extracellular) protein in the open, agonist-bound conformation we recently developed, we evaluate the free energy and the mean first passage time of single-ion permeation using molecular dynamics simulations and the milestoning method with Voronoi tessellation. The results for the wild-type channel provide the first available mapping of the potential of mean force in the full-length α7 nAChR, reveal its expected cationic nature, and are in good agreement with simulation data for other channels of the LGIC family and with experimental data on nAChRs. We then investigate the role of a specific mutation directly related to ion selectivity in LGICs, the E-1′ → A-1′ substitution at the cytoplasmatic selectivity filter. We find that the mutation strongly affects sodium and chloride permeation in opposite directions, leading to a complete inversion of selectivity, at variance with the limited experimental results available that classify this mutant as cationic. We thus provide structural determinants for the observed cationic-to-anionic inversion, revealing a key role of the protonation state of residue rings far from the mutation, in the proximity of the hydrophobic channel gate.

## Introduction

**Figure 1:**
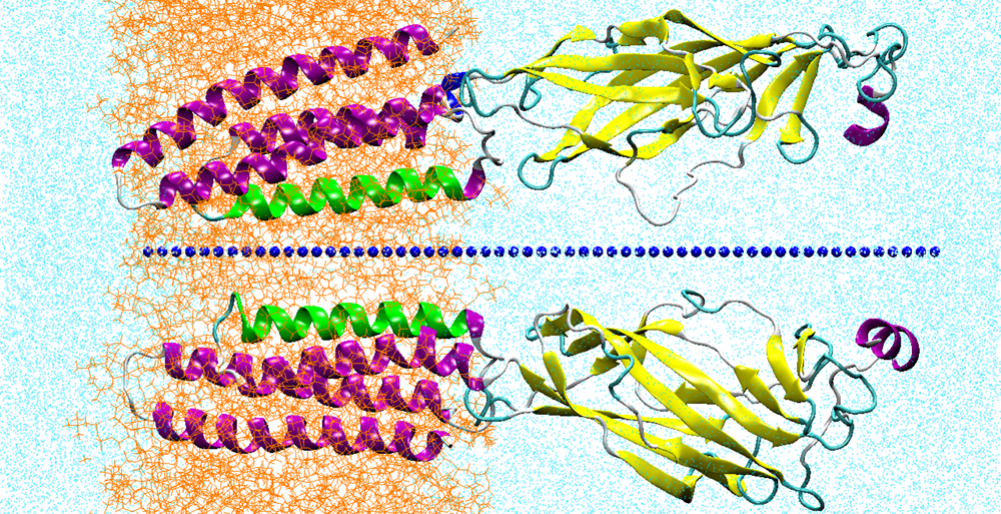
Section of the system simulated: two subunits only are shown, represented in cartoon, for the sake of clarity. M2 helices are colored in green. Blue dots indicate the centers of the Voronoi cells. Lipids are in orange (in line representation); water in cyan (represented with points).

The nicotinic acetylcholine receptors (nAChRs), belonging to the Cys-loop super-family of ligand-gated ion channels (LGICs), are membrane proteins present in neurons and at neuromuscular junctions.^1^ Their overall structure comprises five subunits arranged symmetrically and contains an extracellular domain (ligand binding domain, LBD) and a transmembrane domain (TMD) (see Figure 1). The orthosteric ligand binding site is located in the LBD. The TMD channel pore is formed by the alignment of one helix (M2) from each subunit; it opens following the binding of agonist ligands, while the channel is mostly closed in the resting and in the inactive states, when either no ligand is present or an antagonist is bound as well as in the desensitized, agonist-bound states.

Only over the past few years, high-resolution data became available on the atomic structure of LGICs, in particular, for human and eukaryotic channels. Experimental structures include the human GlyR complexed with strycnine,^2^ complexed with glycine,^3^ the mouse serotonin 5-HT_3*A*_ in the closed form,^4^ and, very recently in two different conformations bound to serotonin,^5^ the desensitized human GABA*_A_*^6^ and *α*4*β*2^7^ structures and the glutamate-gated chloride channel (GluCl) from *Caenorhabditis elegans* (in open and apo-closed conformations^8,9^). Recently, a cryo-EM determination of the *Torpedo californica* acetylcholine receptor in complex with α-bungarotoxin and of the human *α*3*β*4 ganglionic nAChR has also been published.^10,11^

The scarcity of X-ray or cryo-EM data for human nAChRs, however, leads to difficulties in structural annotation of their functional states. In this respect, we recently provided an all-atom model of the human α7 full-length (TMD+LBD) nicotinic receptor, built by a homology model based on high-resolution X-ray templates.^12^ α7 is a homopentameric channel widely expressed in the human brain, involved in regulation of synaptic plasticity and neuronal growth, and related to schizophrenia, Alzheimer’s disease, and even cancer development.^13,14^ Of utmost importance, very recently, α7 has been considered among the nAChRs likely implicated in interaction with SARS-CoV-2 related Covid-19 desease.^15^

In a series of recent papers, we reported on computational structural characterization of α7 in different conformations, putatively associated to four different functional states: open active state, complexed with the full agonist epibatidine;^12^ desensitized;^16^ closed-locked, nonconductive conotoxin-bound state;^17^ and an apo inactive conformation, which recapitulates the conformation of an intermediate state in between the open and the closed states.^17^

In this work, we study the ion permeation mechanism in the putative open α7 channel, with the double aim of (i) further assessing the quality of the open channel model with respect to thermodynamics and kinetics aspects and (ii) elucidating the molecular mechanisms of ions selectivity and permeation.

Ion permeation through transmembrane channels is an event occurring on time scales that are nowadays attainable via standard molecular dynamics (MD) simulations even for large-size systems.^18,19^ However, because of the presence of electrostatic and hydrophobic barriers that slow ion translocation across the pore, accruing enough statistics by observing multiple events can still require prohibitively massive calculations.^20^ Enhanced sampling techniques have been exploited to reconstruct the free energy (FE) landscape underlying the ion translocation process in channels. Potential of mean force (PMF) calculations have been performed for selected eukaryotic LGICs, with different methods and approximations, by limiting the calculations to the TMD only or with implicit description of other regions of the system.

In particular, adaptive biasing force (ABF) calculations have been performed on the 5-HT_3*A*_ channel in its closed state by considering a subsystem composed by the TMD and part of the cytoplasmatic portion of the protein.^21^ Very recently, the sodium and chloride PFM has been calculated with umbrella sampling (US) on the M2 helix bundle of 5-HT_3_ by using polarizable force fields.^22^ Brownian dynamics (BD) calculations have been carried out in GluCl using a dynamic Monte Carlo algorithm,^23^ but in this case, only the ions were explicitly modeled, while the protein channel, the lipid bilayer, and the solvent water were modeled as continua characterized by different dielectric constants. The single-ion PFM profile was calculated using a hybrid approach that combines ABF atomistic calculations in the TMD with a continuum model estimate in large-size pore regions, resulting in barrier values of 10 and 6.1 K*_B_*T for sodium and chloride, respectively, at the selectivity filter.^23^ The PFM of chloride permeation in the human GABA*_A_* receptor has been calculated both in the closed and open states using ABF simulations of the full-length channel.^24^

Extensive BD simulations have been also performed on the prokaryotic homologues of LGICs.^25^ Ion conduction in GLIC has been also investigated by US on the TMD channel only in the wild type^26^ and a mutant (E-2′A).^27^ ABF calculations in conjunction with continuum electrostatic approaches have been performed on the full-length GLIC^28^ at different protonation states of key residues at the selectivity filter.

As for nAChRs, coarse-grained methods as Biology Boltzmann Transport Monte Carlo were applied to models of human muscle nAChR.^29^ Also, US simulations have been performed on a simplified channel pore, composed of a rigid M2 helices bundle from the cryo-EM structure from *Torpedo marmorata*,^30^ claimed in the closed state, and embedded in a bilayer-mimetic slab.^31^ For the constriction pore region, an energy barrier of about 10 K*_B_*T to the permeation of sodium has been obtained. For the same system, however, a United Atoms representation resulted in different values of the conductance.^31^ Also, a definitely lower value of the FE barrier (3 kcal/mol) was obtained in calculations based on a dynamically fluctuating TMD from a homology model of the human α7 nAChR.^32^ BD simulations and continuum electrostatic calculations have been performed on the nAChR from *Torpedo marmorata* to investigate the role of different nAChR domains in ion conduction and selectivity.^33^ The FE profile of sodium has been calculated by US along the M2 pore, in a model of full-length chick α7 based on the cryo-EM structure of *Torpedo*, and embedded in a low-dielectric slab mimicking the lipid bilayer,^34^ with explicit water molecules and ion present.

Overall, results for nAChRs highlight the importance of modeling the channel at the full atomistic level and based on high-resolution reference structures because of the sensitivity of ion permeation to the details of the molecular interactions; furthermore, the role of protein and solvent dynamics in modulating the energetic barriers to ion translocation^29,35^ has to be taken into account.

To provide a refined description of the process in nAChRs, we present in this work the study of ion permeation across the full-length (TMD+LBD), all-atom, human α7 channel model built via homology modeling from high-resolution X-ray crystallography templates and already characterized by us in its conductive and nonconductive forms.^12,16,17,36^ The single-ion PMF (i.e., the PMF of one ion while no other ions are present in the pore) and the ion translocation kinetics are reconstructed in the open channel for sodium and chloride by using the milestoning method with Voronoi tessellation.^37,38^

Milestoning^39^ is a well-established procedure to compute the time evolution of processes such as barrier crossing events or long diffusive transitions between predefined states. The dynamics of the full process is reduced to transition events between intermediates states (the milestones) and the local kinetic information to describe these transitions is computed via short unbiased MD runs between the milestones. In the Voronoi tessellation version of milestoning, introduced by Vanden-Eijnden and collaborators,^37,38^ the system is restrained within Voronoi cells defined in conformational or collective variables space via reflections^37^ or repulsive boundary potentials,^38^ and the edges of the cells are identified as milestones. The necessary kinetic information about the transitions between the milestones is calculated by running short MD simulations of the system, restricted to the cells. The rate matrix of transitions between the milestones, estimated using data collected from the short trajectories, is then used to compute mean first passage times (MFPTs) between milestones. Recent successful applications include ligand binding to myoglobin,^40^ ion permeation through paracellular channels,^41^ and liquid nucleation on surfaces.^42^

In this work, we exploit a tessellation of the configurational space accessible to the ion within the channel embedded in a lipid bilayer and with explicit water. The milestoning approach provides at the same time the free energy profile of ion translocation and the characteristic time (mean first passage time, MFPT) of the full process. Thermodynamics and kinetics results obtained are consistent with the experimentally known cationic nature of the wild-type channel and allow us to identify the structural determinants of ion translocation, i.e., the key residues responsible for the formation of energy barriers and kinetic traps.

In ion channel proteins, several charged amino acid rings along the translocation pathway control ion permeation and the amino acid composition at specific positions has a key role on ionic selectivity and permeation rate.^43−45^ Of particular relevance in LGICs is the highly conserved pore-facing glutamate residues ring, at the position -1′ (on the first N-terminal turn of the pore-forming M2 helices): it is known that the effect of this ring on the amplitude of the single-channel current is larger than other rings of pore-lining charged side chains.^44,45^

Hence, we also investigate here the E-1′ → A-1′ mutant protein via standard MD and calculate the single-ion FE profile and kinetics with milestoning. Results show that the mutation affects ion permeation for both sodium and chloride, in particular, causing inversion of selectivity. Electrophysiology measurements on the E-1′A channel, although limited, indicate that it is still cationic.^46,47^ Inspection of the structural features of the protein allows us to ascribe this difference to the protonation state of the ring of residues at position 20′ far from the mutation. A modification of the 20′ ring should make the mutant channel also cationic while barely affecting the cationic nature of the wild-type channel.

## Computational Methods

### Milestoning with Voronoi Tesselation

Milestoning^39,48^ allows reconstructing the long-time dynamics of a system by exploiting its crossing statistics through a set of hypersurfaces placed along the reaction coordinate. Voronoi-tessellated Markovian milestoning^37,38^ is a version of the method that relies on independent MD simulations confined within a set of cells spanning the reaction coordinate, and uses transition path theory^49^ to obtain the kinetic properties of the full reaction from hitting statistics at the cell boundaries, identified as milestones.

Let us consider a set of *M* points in the CV *z*-space (*z*_1_, *z*_2_, ..., *z_M_*), usually called centers, partitioning the configuration space in *M* Voronoi cells. The Voronoi cell associated to *z_α_* is identified as the region of space where each point is closer to *z_α_* than to any other center *z_β_*. It was shown in ref (37) that the statistical properties of the long-time dynamics of a system can be reconstructed from independent simulations, properly confined within each of the Voronoi cells. More specifically, the confinement must leave unperturbed the dynamical properties of the system when it is in the interior of the cell as well as the probability flux in and out of the cells. Such confinement was realized in ref (37) by using velocity reflections at the cell boundaries. An alternative strategy to confine the CV in the Voronoi cells was proposed in ref (38), and amounts to use half-pseudoharmonic restraining potentials (termed soft walls). This approach was demonstrated to yield the same results of the original one and to allow easier interfacing with the highly optimized, widely used biomolecular simulation MD packages. It requires that portions of trajectories that are transiently out of the cells are discarded in the analysis, but this effect is minimized by proper tuning of the parameters.

In the case of 1D CV used here, *z*, the soft-wall potentials acting in each of the Voronoi cells defined by the *z_α_* points are expressed as
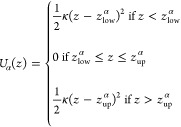
1where *z*_low_^α^ and *z*_up_^α^ denote the edges of the cell and are the midpoints between the center *z_α_* and the adjacent ones.

### MFPT Calculation

By considering the edges of the Voronoi cells as milestones, the dynamics of the system can be reduced to that of a discrete state continuous-time Markov chain in the state space of milestones indices. This amounts to define a rate matrix *q_ij_*, with *i* and *j* indexes of milestones, whose elements are given by *q_ij_* = *N_ij_*/*R_i_*, where *N_ij_* is the number of transitions from *i* to *j* and *R_i_* is the total time that milestone *i* was the last crossed. These factors can be expressed in terms of quantities extracted from the confined simulations, i.e., if we indicate with α the cell index: *N_ij_* = ∑_*α* = 1_^*M*^π_α_(*N*_*ij*_^α^/*T_α_*) and *R_i_* = ∑_*α* = 1_^*M*^π_α_(*R*_*i*_^α^/*T_α_*). Here, *π_α_* is the equilibrium probability of finding the system in cell α, *T_α_* is the duration of the simulation in the cell, and *N*_*ij*_^α^ and *R*_*i*_^α^are defined as *N_ij_* and *R_i_* but for the simulation in α. The PMF associated to the cells is obtained as −*k*_B_*T*ln(*π_α_*),^37^ while the mean first passage times (MFPTs, inverse of the kinetic rates) from any milestone to any other are calculated from the matrix *q_ij_* by solving a linear system, as reported in refs (37) and (38). In our study, we compute the MFPTs between milestones perpendicular to the pore axis from the extracellular to the intracellular mouth of the channel, thus describing the influx of ions into the cell. Since we are interested in the relative permeation time scales of the ions and because there are only one entry and one exit portal in our structure, we neglect the entry kinetics contributions related to bulk concentration and diffusivity that are discussed in ref (40).

### Details of Milestoning Calculations

For the wild-type protein, to construct the milestoning starting configurations, we used the stable open active conformation complexed with the agonist epibatidine, embedded in a lipid bilayer and solvated with water. This system has been already relaxed along extensive MD simulations; details on the system setup and simulation protocol are fully reported in ref (12) and briefly reported in the Supporting Information. The total number of atoms is 142,720 (26,313 protein atoms, 27,360 water molecules, 255 POPC lipids, and 157 ions). As shown in Figure 1, the full-length (TMD+LBD) protein is here investigated along the 110 Å length region connecting the extracellular to the cytoplasmatic limit. To describe ion translocation, we use the ionic coordinate along the direction normal to the bilayer surface. During simulations, the target ion is restrained via repulsive boundary potentials in 57 cells of 2 Å length each, spanning the entire protein axis length. The force constant for the half-pseudoharmonic restraint was set to 100 kcal/mol Å^2^. Such a value, smaller than other spring force constants in the CHARMM force field, is a good compromise between a large value that would better approximate a hard wall but might cause numerical instabilities and a small value that would cause a waste of a large portion of the trajectory (on average, less than 10% of our simulated trajectories lie outside each cell).

The cell size is in the range of the ones used in previous works^40,41^ based on the same method. By monitoring the time evolution of the escape rate elements, we obtained a uniform sampling inside each cell in 30 ns at most, depending on the cell. The cumulative time amounts to 672 ns for sodium and 265 ns for chloride.

All simulations are performed with the NAMD2.12 software.^50,51^ The simulation protocol is the same as for the equilibrium standard sampling simulations^12,16,17^ and is described in the Supporting Information. The milestoning calculations are handled by a home-made Tcl script linked in by the NAMD code, where it has been implemented the calculation of the external forces arising from the soft-wall restraining potentials to confine the ion dynamics inside each cell. The forces act on the ion only, so the time per step in the MD simulations is barely increased. The Tcl script is available from the authors upon request. Note that, for the case of a 1D CV like the one used here, the boundary potentials feature in the NAMD colvar module can also be used.^52^

To rule out multiple pore occupancy in the LBD region, we design an “exclusion sphere”, similarly as in ref (53). The sphere is centered on the LBD center of mass and with 35 Å radius; during the milestoning simulations in each cell, other ions (both cations and anions) are excluded from the LBD lumen with a repulsive flat bottom spherical harmonic restraint with a force constant of 50 kcal/mol Å applied to the ions only when they enter this exclusion sphere (see Figure S1 in the Supporting Information). The “sphere” restraint is also handled by a home-made Tcl script linked in the NAMD code, with a minimal increment of the time/step. A flat-bottom cylindrical restraint with a radius of 20 Å is also applied in the LBD to prevent the ion from drifting too far from the axis of the pore while allowing interactions with the internal channel surface.

As for the mutant, the five E-1′ were replaced with alanine in the structure of the wild-type open channel embedded in the bilayer/water system.^12^ The total number of atoms in the new system is 142,687 (26,286 protein atoms plus five epibatidine molecules, 27,357 water molecules, and 255 POPC lipids). Na^+^ and Cl^–^ ions (160), corresponding to 100 mM solution, were added to neutralize the net system charge. The system was then equilibrated along a 0.5 μs simulation, with the same simulation protocol as for the wild-type system,^12^ detailed in the Supporting Information.

We used the final configuration of this equilibrium run to construct the starting configurations for the milestoning cells. PMF calculations were performed by exploiting a Voronoi tessellation of the region from −20 to 35 Å, spanning the entire TMD. In this case, the ion is restrained via repulsive boundary potentials in 28 cells of 2 Å length each (see blue dots in Figure S1). A single-ion transport mechanism is assumed as for the wild type. No “exclusion sphere” is considered, nor even lateral restraints were applied on the target ion, given that it is well confined inside the pore by the TM channel. We obtained a uniform sampling inside each cell in 26 ns at most, depending on the cell. The cumulative time amounts to 298 ns for sodium and 427 ns for chloride.

## Results

### Wild-Type *α*7

#### The Transmembrane Domain

**Figure 2:**
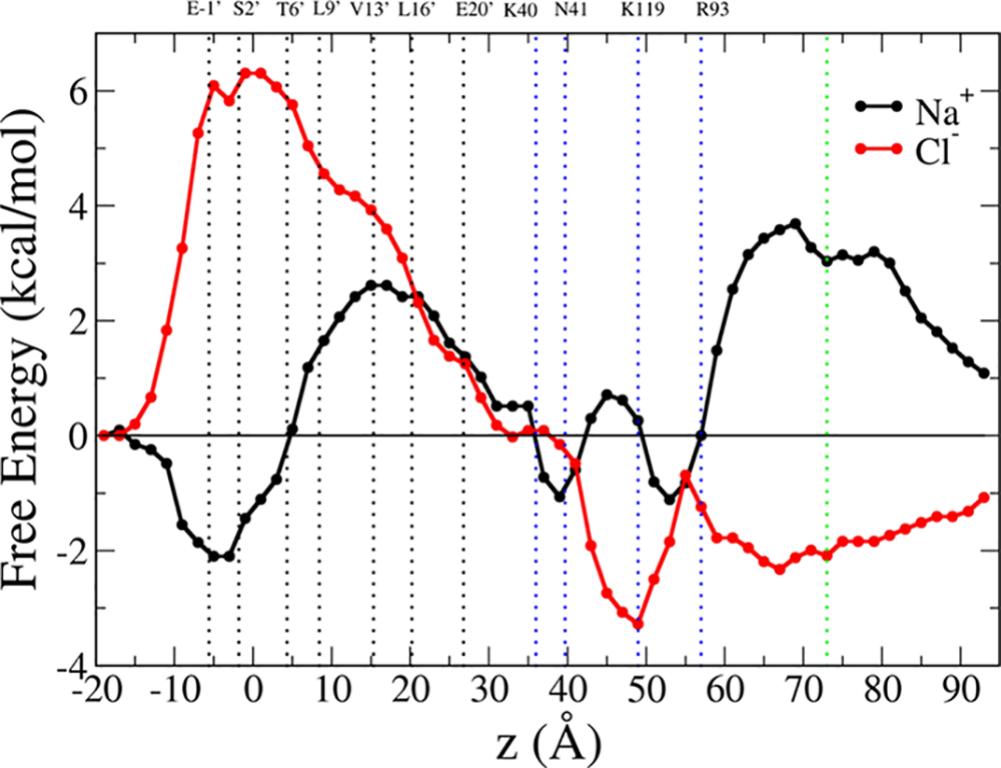
Potential of mean force for the permeation of sodium (black) and chloride (red) through the full *α*7 channel. The curves are shifted along the *y* axis so that their values match at the intracellular end. Positions of M2 pore-lining residues are indicated with black dotted lines; position of key residues in the LBD are indicated with blue dotted lines; the green line indicates the position *z* = 73 Å (center of the Voronoi cell 47), for which the cross-sectional map of the protein electrostatic potential is shown in Figure 3. All key residues are labeled at the top of the graph.

The wild-type *α*7 single-ion PMF profiles are shown in Figure 2 for sodium (black curve) and chloride (red curve). Starting from the cytoplasmatic side, in the case of sodium, the profile exhibits a deep minimum in correspondence of rings of negatively charged/polar residues at the entrance of the pore (E-1′-S2′). A free energy barrier (2 kcal/mol) is located in the middle of the pore in correspondence of the well-known hydrophobic girdle (L9′-L16′). The profile for chloride presents a 6 kcal/mol barrier at the intracellular end of the channel, mostly due to the ring of negatively charged glutamate residues (E-1′). This is the largest barrier for chloride in the full-length profile, indicating that the E-1′ ring plays the dominant role in ion selection in the human *α*7 channel. A shoulder of about 4 kcal/mol appears at the hydrophobic girdle, fully consistent with results in the literature.^28,32,54^ Indeed, it is well known that the hydrophobic gate region plays an important role in distinguishing cation-selective from anion-selective LGICs.^28^ In wild-type, cation-selective channels, hydrophobic residues generate a higher energy barrier for the permeation of anions, while hydrophilic pore-lining segments are found in anion-selective LGICs, which serve as a partial hydration shell around the permeating Cl^–^ ions, facilitating their passage.^55^ The value of about 2 kcal/mol found for the sodium ion indicates that the channel is indeed in an open conformation, physiologically associated to cation permeation, as already observed by our previous standard MD simulations.^12,16,17^

Both profiles fall to zero kcal/mol in correspondence of the LBD–TMD interface. An estimate of the single-channel maximum conductance based on the TMD portion of the single-ion PMF profiles^31,56^ gives 0.17 pS for chloride and 1.7 pS for sodium, i.e., 10 times higher than for the anion. The result is consistent with preferential selectivity of wild-type *α*7 for cations.

#### The Ligand Binding Domain

In the LBD, sodium and chloride PMF profiles are symmetric and arise from repulsion/attraction of pore facing charged/polar residue rings, respectively. A small free energy barrier for the positively charged sodium ion is present, in the range of 40–60 Å. It arises from the presence of positively charged arginine/lysine and polar asparagine rings in the *β*1–*β*2 tip, in the *β*4–*β*5 loop, and in *β*10 (K40-N41-R93-K119). On the contrary, the interaction with these rings provides a deep minimum at −4 kcal/mol for chloride. The sodium profile presents another wide barrier in the range of 60–90 Å of about 4 kcal/mol. In contrast, we observe a shallow minimum (−2 kcal/mol) for the chloride ion in the same range.

**Figure 3:**
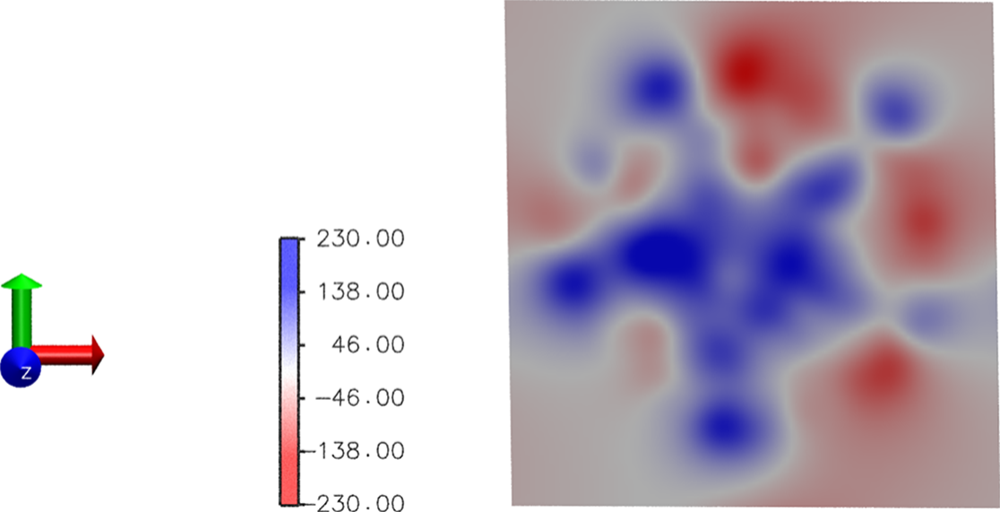
Cross-sectional map of the protein electrostatic potential at *z* = 73 Å. The map has been calculated using the PMEpot plugin in VMD^57^ (see the Supporting Information). At *T* = 310 K, one PMEpot unit of electrostatic potential is equivalent to 27 mV.

Results are fully consistent with the electrostatics in the channel. As an example, Figure 3 shows a cross-sectional map of the protein electrostatic potential at *z* = 73 Å, i.e., well inside the shallow minimum of the chloride PMF or at the top of the sodium barrier (indicated with the green dotted line in Figure 2), averaged along the chloride milestoning trajectory. Overall, the protein electrostatic potential is positive inside the pore lumen in this portion of LBD, determining the PMF profiles for both chloride and sodium.

**Figure 4:**
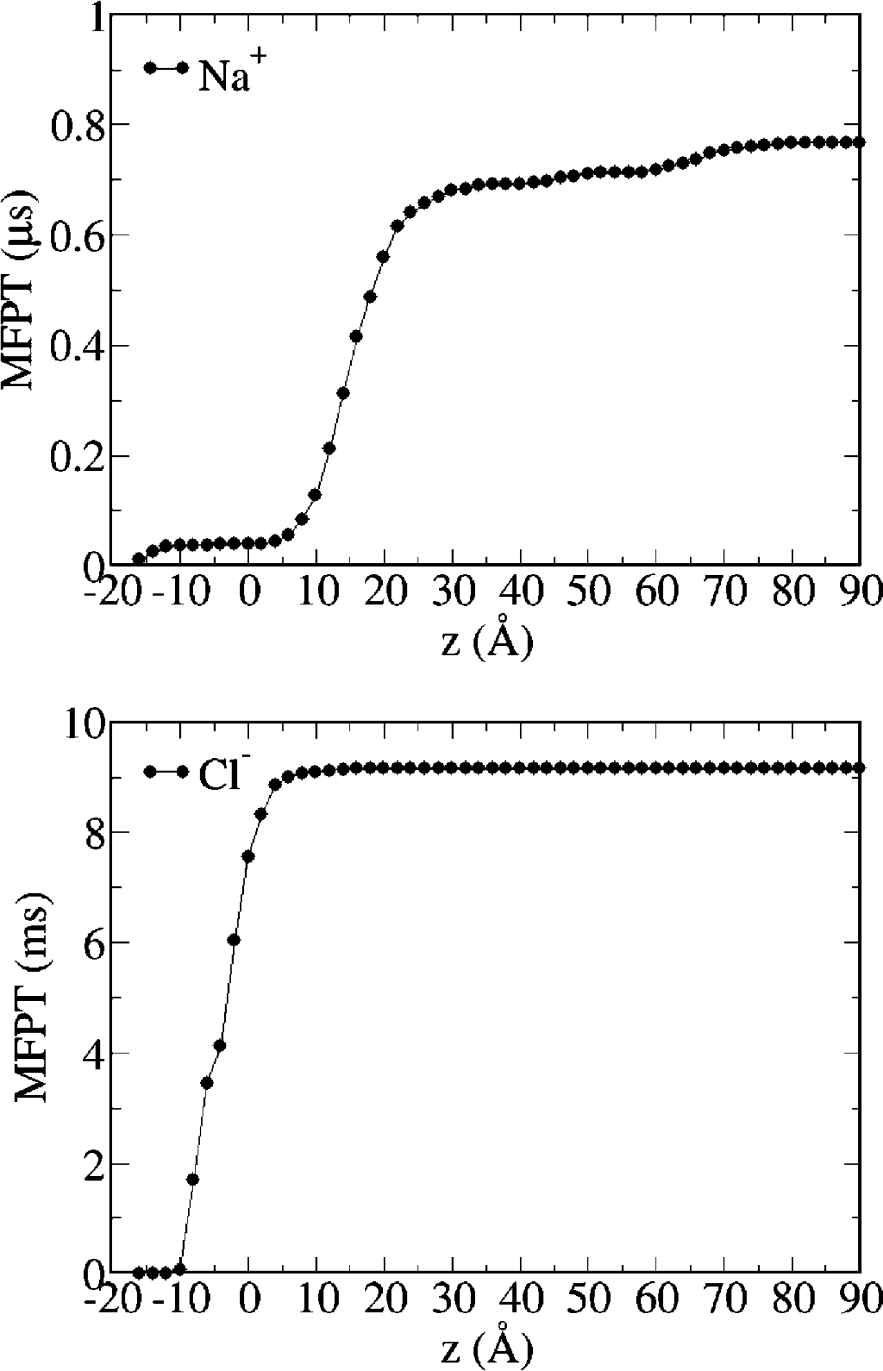
Mean first passage times from all milestones to the intracellular mouth milestone. Upper panel: sodium; lower panel: chloride.

Milestoning MFPTs for ion permeation from the extracellular to the intracellular side are shown in Figure 4. The MFPT to traverse the full channel is smaller for sodium than chloride (0.784 μs and 9.165 ms, respectively), consistent with the experimentally determined cationic nature of wild-type *α*7. Moreover, results indicate that the FE barriers in the TMD play the major role in ion permeation, in agreement with results from simulations on GLIC,^25^ as the MFPTs are only slightly reduced along the LBD. Consistent with the hypothesized role for the LBD in maintaining a high concentration of cations at the mouth of the pore, Figures S7 and S8 (upper panel) show that the vestibules of the receptor below and above the TM pore are markedly electronegative, thus providing an environment to stabilize cations and increasing their local concentration. Within the pore, cations are further stabilized by interactions with ionized side chains in the first turn of the M2 helices (e.g., E-1′).

**Figure 5:**
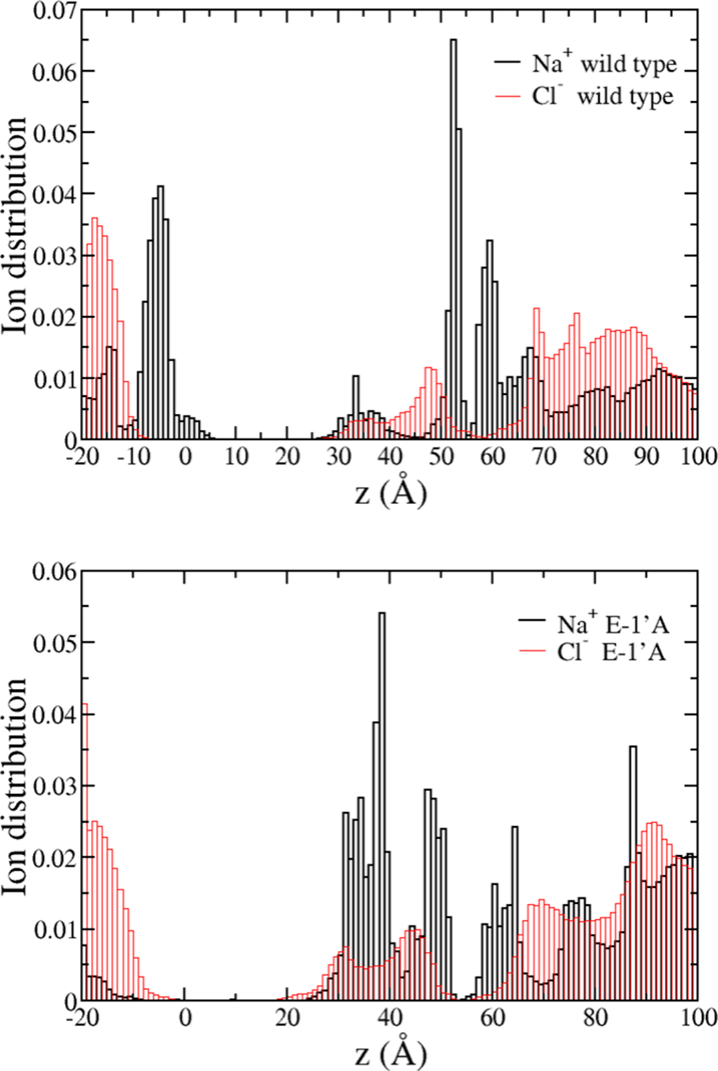
Ion dwell distribution in wild type (upper panel) and mutant (lower panel) systems. Black bars: sodium; red bars: chloride. To construct the histograms, the channel is divided into 120 thin sections, and the average number of ions in each section is calculated along the last 200 ns segment of both equilibrium simulations.

Ion dwell distributions calculated along the standard MD trajectories (Figure 5 and see also in ref (12)) are in full correspondence with the PMF profiles (note that, for the LBD, these refer to multi-ion densities, while the PMF is always single-ion). A peak is observed for sodium density in correspondence of the minimum located at −5 Å, and no event of sodium translocation is observed across the pore up to about 30 Å on the hundred of nanosecond time scale. Sodium is persistent in correspondence of secondary minima (−1 kcal/mol) of the FE profile, located in the ranges of 35–40 Å and 50–55 Å respectively, while a peak for chloride is present at about 50 Å, in correspondence to the FE well. In the range of 60–90 Å, the ion dwell times are in full agreement with the presence of the wide FE barrier for the sodium, in parallel to the shallow free energy minimum in the chloride FE profile.

### The E-1′A Mutant *α*7

To better understand the role of the E-1′ ring in ion selectivity, we simulated the E-1′A full-length *α*7 embedded in a POPC bilayer/water system. The new system was relaxed along a 0.5 μs MD simulation, with the same simulation protocol as for the wild-type conformation,^12^ detailed in the Supporting Information.

To assess the overall stability of the mutant conformation, we evaluated the *C_α_* root mean square deviation (RMSD) from the initial configuration and the root mean square fluctuations (RMSFs) with respect to the structure averaged over the last 200 ns of the run. Results are shown in Figures S2 and S3 in the Supporting Information, separately for the LBD and the TMD of the five subunits. The time evolution of the RMSDs points out that, averaging over the five subunits, the conformation of the channel is stable on the reported time scale both at the LBD and TMD level. The *C_α_* RMSF profile along the protein chain is quite similar to that obtained from previous simulation of the wild-type model.^12^ Peak locations are conserved, in particular, the *β*2–*β*3 loop, A-loop, Cys-loop, F-loop, and the C-loop (that caps the epibatidine molecules) so as the loops connecting M1-M2 helices in the TMD. We observe a large peak in the region of helix M4 around residue 300, a known consequence of the structural uncertainty associated to the loop M3-M4.^58^ The RMSD curve relative to the subunit P5 (yellow curve) is higher than the others. This behavior is mostly brought about by motions of the F and C loops in the subunit, as testified by the corresponding peaks in the RMSFs profiles in Figure S3.

Figure S4 in the Supporting Information shows the pore radius profile along the channel axis in the TMD (blue curve, structure averaged over the last 200 ns of the trajectory). Compared with the wild-type structure (red curve, see also in ref (17)), the E-1′A mutation results in a wide profile at the intracellular end due to the absence of the five glutamate residues pointing their side chain toward the center of the pore, as in the wild-type channel. Indeed, the alanine side chains are smaller compared with the bulky glutamate side chains, and given their hydrophobic nature, they do not point toward the pore center but are preferentially oriented tangentially to the M2 pore lumen, allowing more room to the chloride ion. Results are in full agreement with the ones on GLIC for the same mutation (see Figure 2 in ref (25), comparison between wild-type GLIC and mutant (GLICM) structures).

Widening of the pore actually corresponds to a sizable mutual rearrangement of the M2 helices, which undergo both a polar and lateral tilt (of about 4°) with respect to the native conformation (Figure S5 in the Supporting Information, upper panels). This rearrangement is compatible with pore widening and at the same time avoiding pore closure at the top entrance, which would lead to pore dehydration and increase of the barrier to ion influx. M2 tilting transmits to the LBD resulting in an overall quaternary twist and “blooming” of the LBD with respect to the TMD^17^ (Figure S5 in the Supporting Information, lower panels).

Figure S6 shows the time evolution of water count in the pore lumen lined by the M2 helices and in a region of 10 Å length, centered at the V13′ site, well inside the hydrophobic girdle. Results on the hundred of nanosecond time scale are similar in the wild type and mutated proteins. The number of water molecules in the full channel, 30 Å long, is stationary at ∼105 along the whole simulation of both conformations. In the region Leu9′-Leu16′, the average number of water molecules is about 30 in both structures. By general consensus, pore hydration observed in standard equilibrium MD simulations can be used as a reliable determinant for ion permeability.^59^ Therefore, results indicate that, also in the mutated structure, an open, fully hydrated, pore is present, which allows ion permeation across the membrane.

PMF calculations were performed on the full-length equilibrated structure by exploiting a Voronoi tessellation of the region −20 to 35 Å spanning the entire TMD only as we assume that the effects of substituting the residue at -1′ are restricted to the TMD. Indeed, the protein electrostatic map calculated on the mutated structure (averaged over the last 200 ns portion of the equilibrium trajectory, see Figure S7, lower panel, in the Supporting Information), is very similar in the LBD to the wild-type map while markedly different in the TMD. The same holds for the ion dwell distributions, shown in Figure 4, lower panel, which clearly show how the mutation strongly affects the ion concentration in the TMD, with minor differences in the LBD. This agrees with results from simulations on nAChR from *Torpedo marmorata*,^33^ where after substitutions in the TMD, the electrostatic potential in this region was found higher than in the wild-type protein and almost unaltered in the LBD.

**Figure 6:**
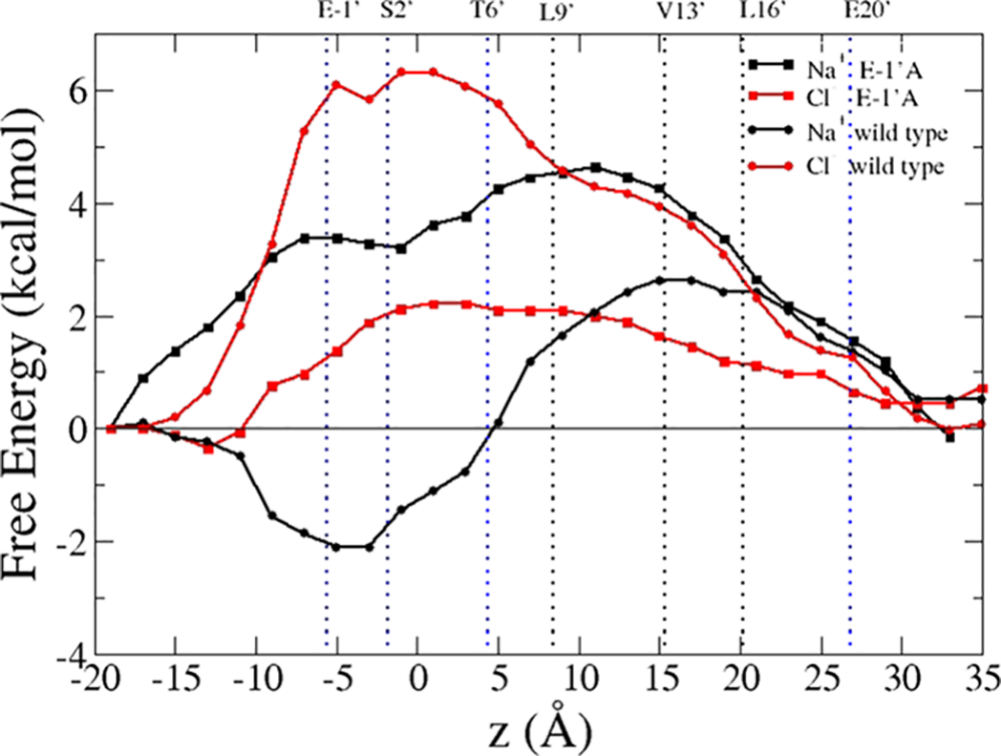
Potential of mean force for the permeation of sodium (black squares) and chloride (red squares) through the TMD in the E-1′A mutant compared with the wild-type channel (sodium, black circles; chloride, red circles). The curves were shifted along the *y* axis so that their values match at the intracellular end. Positions of M2 pore-lining residues are indicated with black (hydrophobic) and blue (polar) dotted lines and labeled at the top of the graph.

The single-ion sodium and chloride PMF profiles in the TMD of the mutated protein are shown in Figure 6 and compared with the ones of the wild-type protein, already shown in Figure 2.

**Figure 7:**
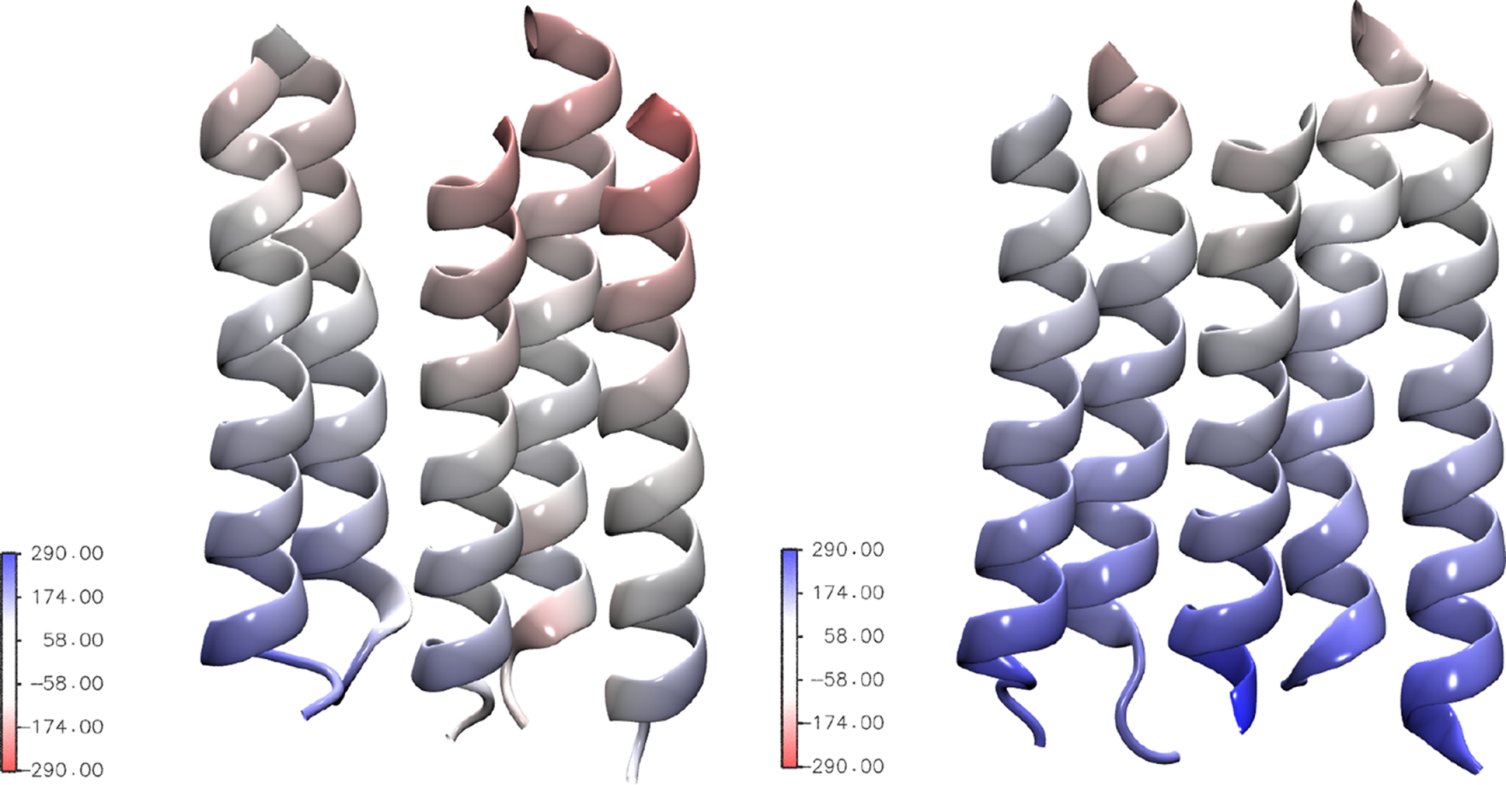
M2 helices colored according to the protein electrostatic potential calculated with PMEpot,^57^ averaged along the chloride trajectory in the window centered at the position of VAL13′. One PMEpot unit of electrostatic potential is equivalent to 27 mV. Left panel: wild type; right panel: E-1′A mutant.

The effect of neutralizing the E-1′ ring with alanines is very different for the two ions and in the opposite direction. The kinetic trap for the sodium located in the range of −10 to 5 Å disappears, while the barrier at the hydrophobic girdle increases from 2 to 4 kcal/mol. On the contrary, the chloride PMF exhibits a reduction of about 4 kcal/mol with respect to the wild-type profile in the barrier region −10 to 5 Å. The FE profiles are consistent with the characteristics of the protein electrostatic potential and with the ion dwell distributions evaluated along the equilibrated mutant trajectory. The electrostatic potential of the pore is more positive than in the wild-type case, as better shown in Figure 7, where the five M2 helices lining the pore lumen are shown, colored according to the protein electrostatic potential averaged along the trajectory of the target chloride at *z* = 16 Å, i.e., in correspondence of the V13′ site. A full map of the electrostatic potential of the intact channel, averaged along the same restricted simulation, is also shown in Figure S8.

As for the ion dwell time distributions (Figure 5, lower panel), the peak observed for sodium in the wild-type case has disappeared in the mutant, consistent with the FE barrier encountered at the intracellular mouth. As in the wild-type, no event of sodium translocation is observed across the pore up to about 30 Å. Sodium persistence in observed in the LBD where the dwell time histogram is almost comparable with the wild type. Chloride accumulates from the TMD/LBD interface down to 20 Å (which approximately correspond to the L16′ position) and on the intracellular side in the range −10 to 0 Å at the extremes of the small energy barrier.

An estimate of the single-channel conductance based on the single-ion PMF^31,56^ gives 3.3 pS for sodium. Compared with the wild type (1.7 pS), the sodium conductance is slightly increased. Something similar has been found in GLIC simulations,^28^ and in analogy with the GLIC case, this results could be justified by the fact that, when a partially negatively charged E ring is present, the binding site strength at the -1′ site is higher, decreasing the rate for a cation to dissociate from this binding site.

As a major result, the conductance is 45 pS for chloride, i.e., about 10 times that for sodium. Compared with the wild type, it seems that the selectivity has been inverted, the ratio of conductances being of the same order than the sodium/chloride ratio in the wild type.

**Figure 8:**
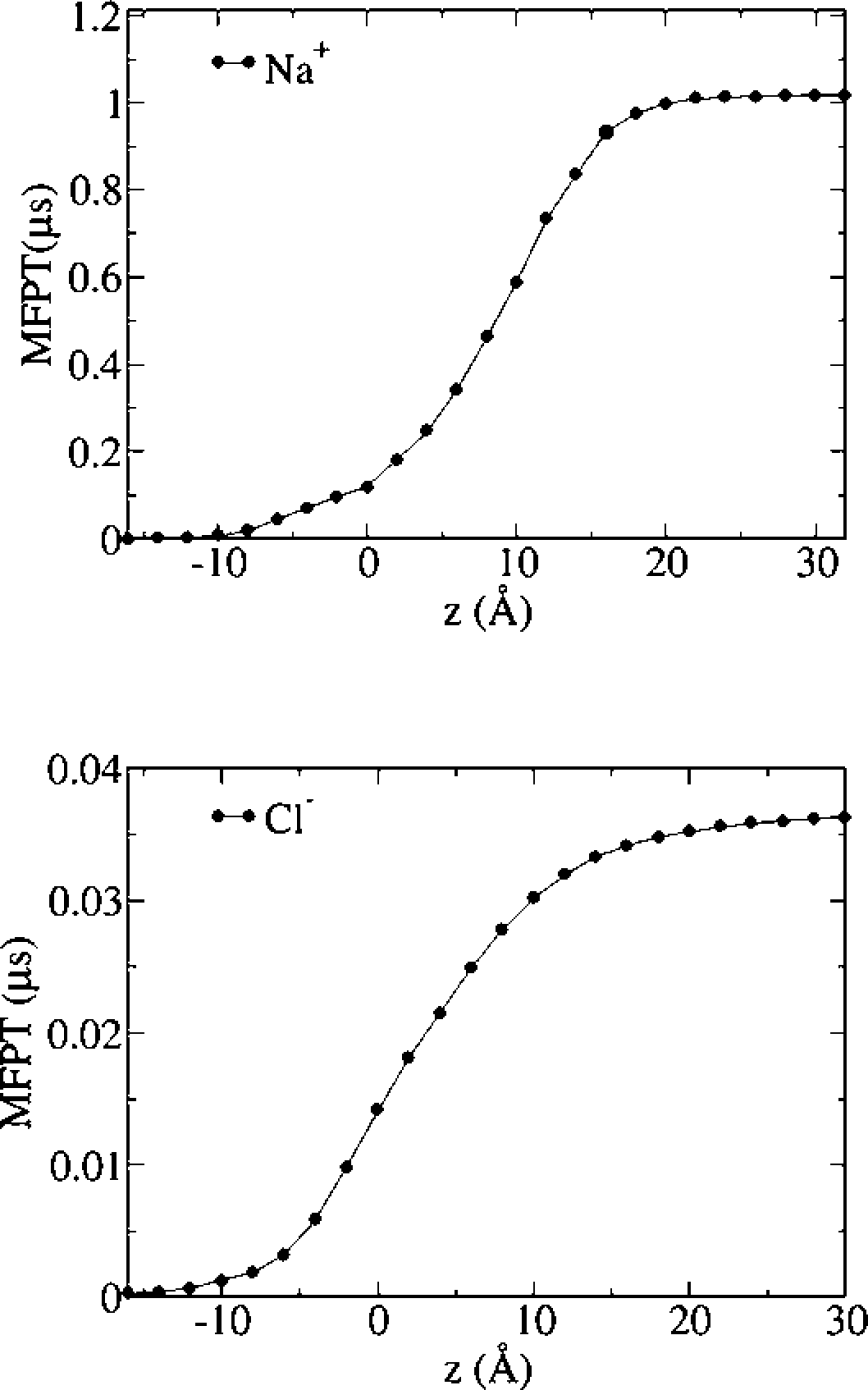
Mean first passage times from all milestones to the intracellular mouth milestone. Upper panel: sodium; lower panel: chloride.

Figure 8 shows MFPTs from all milestones to the intracellular mouth milestone for sodium and chloride. The MFPT from the TMD/LBD interface milestone are 1 μs for sodium and only 36 ns for chloride, indicating that the E-1′A mutation reverses the selectivity of the ion channel in our simulations, as already evidenced by the PMF profiles for both ions.

## Discussion

Simulation methods at different levels of resolution provide valuable tools in structural and functional annotation of the various physiological states of channels of the LGIC family.^59^ This work follows a previous structural assessment of a model of the open state of the human *α*7 nicotinic receptor, complexed with a full agonist, built via homology model based on high-resolution X-ray templates, and relaxed along extensive MD simulations.^12,16,17^ Here, it is shown how the model indeed captures a structure in the conductive state by investigating the mechanisms of ion permeation, both in the wild type and in the mutant structures.

Ion translocation is a rare event associated with crossing high FE barriers encountered by the ion in its path from the LDB/TMD interface toward the intracellular side. Even in the case of an open conductive channel, microsecond-time scale unbiased MD simulations, in the absence of external electric fields, would fail in collecting sufficient statistics of ion crossing events.^18,20^ To circumvent this issue, we exploited advanced sampling techniques to reconstruct the FE profiles of both sodium and chloride translocation across the full-length channel. Milestoning with Voronoi tessellation has been implemented to obtain an estimate of both the FE barriers and the MFPT of the full process, with a cumulative computational time on the hundred of nanosecond time scale.

This is the first time the complete 1D PMF profile is calculated by atomistic simulations on the full-length (TMD-LBD) human *α*7 channel in the wild-type conformation. As for the extracellular domain, it is shown how the contribution to the ion translocation mechanism arises from electrostatic interactions with alternating rings of charged/polar side chains, resulting in symmetric profiles for sodium and chloride. The effect of the LBD on the kinetics of translocation is minor, while it clearly helps in concentrating the permeant ion at the mouths of the TMD, in agreement with results from simulations on GLIC.^25^ The FE barriers found in the TMD are in agreement with experimental and simulative results on nAChRs and other channels of the LGIC family and play the major role in ion permeation in nicotinic receptors.

The sodium maximum conductance calculated on the basis of the TMD PMF profiles is 10 times higher than for chloride, confirming that the wild-type *α*7 is indeed a cationic channel. However, the cation value is lower than the experimental value for open conductive nAChRs (30–50 pS, single-channel measurements under physiological conditions of the (open) Torpedo nAChR).^46,60,61^ Despite the fact that a direct comparison between simulations and experiments is very difficult due to the different operating conditions, we speculate that the discrepancy found could be attributed to the charge state of the E20′ ring, which, in our case, are all protonated. Indeed, mutagenesis experiments showed substantial changes in conductance after mutating this specific ring of charged residues,^60^ and in particular, it has been reported that consecutive mutations from E to A at position 20′ (e.g., neutralizing the E charges) led to a progressive reduction in conductance.^46,60,61^

To further validate our model, we investigated a mutant channel bearing the E-1′A substitutions. Our results point out an inversion of selectivity from cationic to anionic. A similar result has been found in other channels of the LGIC family and, in particular, in several homomeric cationic/anionic LGICs, which all share a glutamate at the -1′ position. As an example, simulations of the cation-selective GLIC, where the charges on the intracellular E ring were decreased from 0.6 e to 0 to mimic the E-1′A mutation,^25^ showed a ratio of permeating chloride/sodium ions of about 10, thus revealing a switch to anion selectivity. On the experimental side, the same mutation in the 5-HT_3*A*_R was sufficient to convert cation into slight anion selectivity.^62^ On the other hand, in the homomeric anionic Gly receptor, the reverse A-1′E mutation alone was able to provide cation-selective channels.^63^ The double mutation Δ290/A291E in GABA reversed the charge selectivity from anionic to cationic, while the single mutation A291E allowed mixed cation and anion permeability.^64^

Results from these experimental studies are at odds with results from experiments on the *α*7, where the single E-1′A mutation had no effect on selectivity, while at least a double substitution E-1′A and at the hydrophobic girdle (V13′T), plus the insertion of a proline just before the glutamate, was needed to make the channel anionic.^60,65−71^ In Jensen et al.,^72^ the question if this discrepancy is due either to a fundamental difference between the *α*7 receptor and the other LGICs or perhaps to different experimental conditions has been raised.

We then sought to reveal possible sources of the discrepancy between our simulation and the experimental result on the mutant. First, by careful inspection of the trajectories, we excluded the co-presence of other chloride ions in the milestoning trajectories restricted in the TMD cells, which would screen anion–residue interactions inside the pore. It is worth noting that co-presence in the TMD was also not observed for sodium or for both ions in the wild-type system.

As shown in Figure 7 and Figures S7 and S8 in the Supporting Information, in our simulations, the mutation causes a change of the electrostatic potential inside the TMD, yielding a more electropositive environment favorable to the anion. As recently reported,^73^ besides the change in electrostatic interactions due to the mutation, a relevant role in determining ion permeation is attributed to the features of the ion hydration shells and, most importantly, to the residue dynamics inside the pore. This in particular when asymmetry is found between the sodium and chloride PMF profiles, as also suggested from results of GLIC simulations.^27^ Therefore, the features of sodium and chloride permeation in our mutant structure at the hydrophobic girdle located in the 10–20 Å range deserve some detailed inspection.

**Figure 9:**
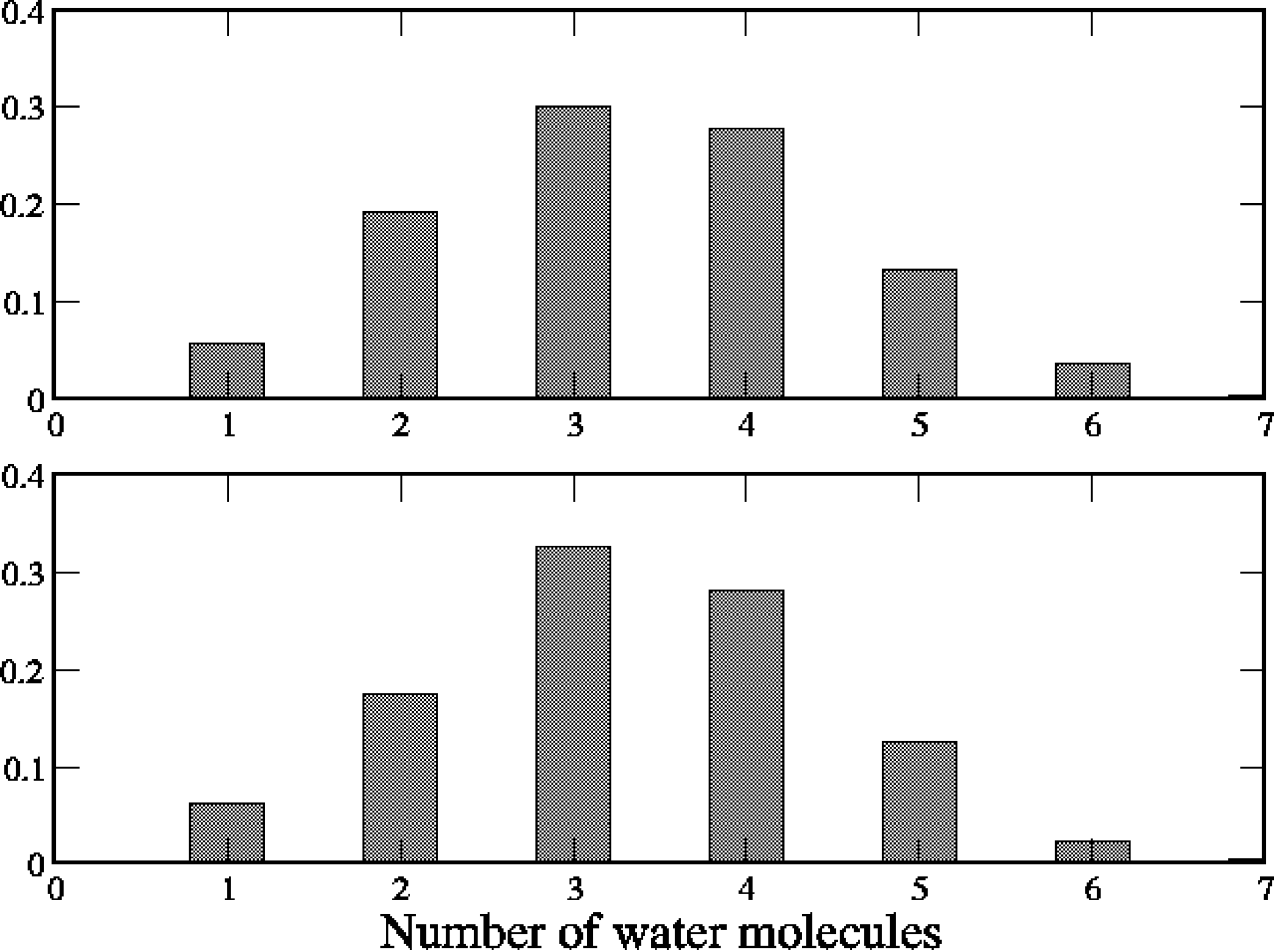
Distribution of the number of water molecules oxygen atoms within 3.2 Å from the chloride ion, calculated from trajectories in the Voronoi cell #18 (upper panel) and cell #19 (lower panel).

Figure 9 shows as an example the distributions of the number of water oxygen atoms in the first hydration shell of chloride (radius set to 3.2 Å^74^) along the restricted simulations in correspondence of position V13′ (Voronoi cells #18 and #19). We observe several events where only one-to-three water molecules are found within the chloride hydration shell, although the pore is not constricted in this region (see Figure S4 in the Supporting Information). In principle, partial ion dehydration would create a barrier to ion permeation when not compensated by favorable ion interactions with protein residues. Indeed, a careful analysis of the system trajectories reveals that partial desolvation of the ion at V13′ is compensated by interaction with some side chains pointing toward the pore center. This is the case of the SER10′ ring located between L9′ and V13′ (data not shown) and the E20′ rings located on the top of the hydrophobic girdle.

The E20′ side chains (protonated in our system) were found able to fluctuate among different conformations along the trajectory, as evidenced in Figure S9, where time series of the C–Cα–Cβ–Cγ angle are shown for E253, E909, and E1237, belonging to three different subunits (milestoning trajectory restricted at V13′). The torsion angle fluctuates between 60° and 180° (up- and anti-conformations, respectively) corresponding to the OH groups pointing toward the center of the pore. These conformations are the ones mostly visited when the glutamate side chains are protonated.^27^

**Figure 10:**
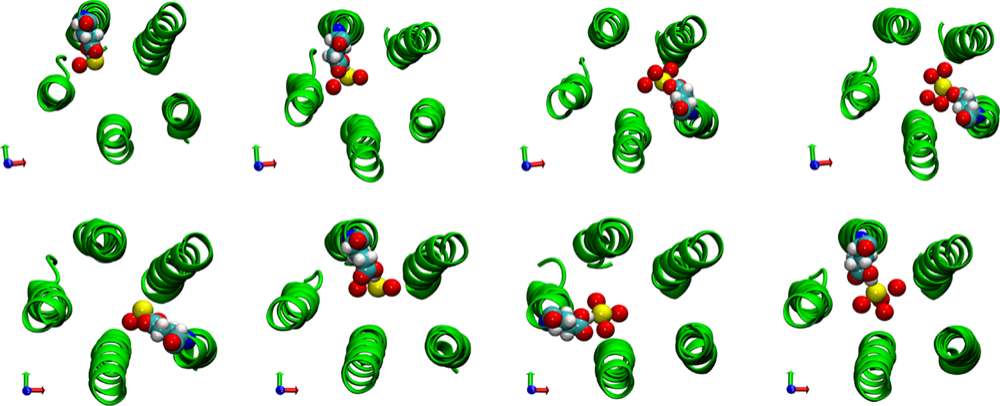
Trajectory snapshots depicting the translocation of chloride ion at position 13′ along the pore (from trajectories in the Voronoi cell #18 (upper row) and cell #19 (lower row)). For the sake of clarity, only the M2 helices are represented (in green, cartoon representation). Yellow spheres: the chloride ion; red spheres: ion hydration shell water oxygen atoms (from left to right: one to four water molecules). E20′ residues in vdW (van der Waals) representation.

In Figure 10, corresponding snapshots of the system are shown, clearly indicating how the ion hydration shell is partially substituted with the E20′ hydroxyl groups. This happens when side chains are in anti-conformation (C–Cα–Cβ–Cγ = 180°). In this case, the E20′ act as surrogate water molecules, facilitating partial hydrated anions to pass. A similar behavior has been recently reported in the case of a single mutation in the muscle acetylcholine receptor,^75^ which was found able to reverse the selectivity. In that case, the inversion was justified on the basis of a stabilization of the permeating chloride ion by arginine residues compensating partial desolvation.

Remarkably, no similar events are observed neither in the sodium case nor in the wild-type case for both ions. Hence, our simulations suggest how interaction with rings located below and above the hydrophobic girdle could assist the chloride translocation, contributing to offset the ion dehydration penalty and leading to a lower free energy path through the hydrophobic region.

If we assume that the lateral chains of E20′ were charged but still able to interact with chloride within 3.2 Å, a simple estimate of the electrostatic interaction energy between the chloride ion and the E20′ carboxyl oxygen would result in about 1.5 kcal/mol, assuming a dielectric constant inside the channel equal to 60.^33,76^ Hence, we can speculate that chloride interaction with one to three different subunits could contribute to increase the PMF barrier of the chloride at the hydrophobic girdle. At variance, a charged E20′ ring would modify the PMF profiles we obtain for the wild-type structure but without changing the selectivity since, while increasing the barrier for chloride, it would make the TMD more attractive for sodium.

## Conclusions

A reconstruction of the PMF profile for ion translocation across the human α7 channel is presented both in the wild type and in the E-1′A mutant by exploiting the milestoning with Voronoi tessellation method, which provides, at the same time, both the free energy barriers and the MFPT of the full process. In the wild-type conformation, cation and anion PMF profiles are calculated by full-atom simulations on the full-length (TMD+LBD) channel for the first time. Results are quite similar to the ones obtained from simulations of ion permeation in the full-length GLIC channel,^28^ which however combined atomistic ABF simulations with coarse graining approaches to investigate the extracellular domain. In the mutant case, ion PMFs are calculated by taking into account the full-length channel in atomistic simulations but limiting the tessellation of the ion coordinate to the TMD, where most of the effects of the mutation are supposed to occur, consistent with what we observe in standard MD simulations.

A comparison with results on the mutant points out the importance of the E residues at -1′ in ion permeability, selectivity, and as a cation binding site in the LGIC receptors. The data shown here point out that an anionic channel has been produced by neutralization of the -1′ site alone. This result, combined with the results from mutagenesis experiments on several channels of the LGIC family,^62−64^ attributes to the E-1′ mutant the most important role in ion selectivity.

Structural determinants for the observed inversion of selectivity are given. Results found in our simulations suggest how chloride interaction with polar side chains of rings located below and above the TMD hydrophobic girdle could give the same effect as neutralizing the hydrophobic region, therefore determining the inversion of the ion selectivity as experimentally found in α7 following a double mutation (E-1′A plus V13′T). This support a previous suggestion that, while the electrostatics has a dominant role in ion selectivity and permeation, both the ion hydration and most importantly the dynamics of residues lining the pore should be taken into account to rationalize the PMF results.

Results presented in this work will be useful to study other mutations associated to pathological α7 phenotypes and to provide a molecular basis for the receptor malfunction.^77^

Last but most importantly, the human α7 nicotinic receptor has recently raised attention in view of a possible implication of this protein in Covid-19 desease.^15^ As suggested by the authors, further work is needed to unravel the relationships between ACE2 and nAChRs in the nervous system. In this respect, a reliable full-atomistic model that recapitulates the open conductive state, as the one presented in this work, constitutes a valuable proxy for studying this interaction at a molecular level.

## Supporting Information Available

Schematic representation of the TMD-LBD channel, where the centers of the Voronoi cells and the ion “exclusion sphere” in the LBD are sketched; *C_α_* root mean square deviation (in Å) of individual subunits calculated from the starting conformation in the E-1′A mutant, along the equilibrium 0.5 μs trajectory; *C_α_* root mean square deviation (in Å) of the LBD and TMD calculated from the starting conformation in the E-1′A mutant, along the equilibrium 0.5 μs trajectory; *C_α_* RMSFs (in Å) in the individual subunits with respect to the E-1′A mutant structure averaged over the final 200 ns of the 0.5 μs trajectory; pore profile radius in the wild type and E-1′A mutant; distributions of the M2 polar and azimuthal tilt angles, quaternary TMD/LBD twist, and LBD blooming; time evolution of water molecules inside the pore in the wild type and E-1′A mutant; protein electrostatic maps in the wild type and E-1′A mutant, averaged over the equilibrium trajectories; protein electrostatic map in the wild type and E-1′A mutant, averaged along the trajectory in Voronoi cell #18‘ and time evolution of the torsion angle of E20′ residues belonging to subunits P1, P3, and P4 (PDF)
