# Supplementary material for: Thermodynamics and Kinetics of Ion Permeation in Wild-Type and Mutated Open Active Conformation of the Human α7 Nicotinic Receptor
Source: J Chem Inf Model. 2020 Aug 17;60(10):5045–56. doi: 10.1021/acs.jcim.0c00549 (PMC8011927; doi:10.1021/acs.jcim.0c00549)
Supplement: Supplementary file 1 — ci0c00549_si_001.pdf [file ci0c00549_si_001.pdf]

Supporting Information for: Thermodynamics and kinetics of ion permeation in wild-type and mutated open active conformation of the human α7 nicotinic receptor

Grazia Cottone,*,† Letizia Chiodo,‡ and Luca Maragliano¶,§

†*Department of Physics and Chemistry-Emilio Segrè, University of Palermo, Viale delle Scienze Ed. 17, 90128 Palermo, Italy*

‡*Department of Engineering, Campus Bio-Medico University of Rome, Via Á. del Portillo 21, 00128 Rome, Italy*

¶*Center for Synaptic Neuroscience and Technology (NSYN@UniGe), Istituto Italiano di Tecnologia, Largo Rosanna Benzi, 10, 16132 Genova, Italy;*

IRCCS Ospedale Policlinico San Martino, Largo Rosanna Benzi, 10, 16132 Genova, Italy

§*Present address: Department of Life and Environmental Sciences, Marche Polytechnic University, Via Brecce Bianche, 60131, Ancona, Italy*

E-mail: grazia.cottone@unipa.it

Simulation protocol for standard MD simulations and milestoning simulations in the Voronoi cells

All MD simulations were performed with the NAMD2.12 package,[1,2] with the CHARMM27 force field for proteins, ions, and water and the CHARMM36 force field for phospholipids. Topology for the epibatidine was generated by Antechamber;[3] ligand partial atomic charges

were obtained by us from ab initio calculations with Gaussian 03.[4] All simulations were performed in the NPT ensemble at T = 310K and P = 1atm.[5] Periodic boundary conditions were applied, with particle-mesh Ewald long-range electrostatics,[6] using a grid spacing of 1 Å along with a sixth order B-spline charge interpolation scheme. A cutoff of 1.2 nm for Lennard-Jones potential, with a smooth switching function starting at 1.0 nm, was used. Bonds involving hydrogen atoms were constrained to their equilibrium length using the SHAKE/RATTLE algorithm,[7] with time step of 1 fs.

For the wild type case, the system set up and the equilibration protocol of the protein/lipid bilayer/water system are the ones fully described in ref.[8] We remind here that protonation state of ionizable residues at physiological pH (7.4) was predicted by using the web-based implementation of a method for pKa calculations based on continuum electrostatic model[9] (http://biophysics.cs.vt.edu/H++)[10]), by careful choice of the values for the dielectric constants of the protein domain interior/exterior, depending on the environment (solvent water or lipid bilayer) to which the domain is exposed. As result from these calculations, three out of five E-1′ residues were found negatively charged, while the five E20' were found all protonated.

The total number of atoms in the wild type system is 142720 (26313 protein atoms including the five epibatidine molecules, 27360 water molecules, 255 POPC lipids, 77 Na^+ and 80 Cl^- ions, corresponding to 100 mM solution, added to neutralize the net system charge). Starting from an equilibrated configuration of the wild type protein/bilayer/water system, we just replaced the five glutamate residues at position -1′ in the protein with five alanine residues, by keeping unaltered the protonation state of the other residues. The total number of atoms in the new system is 142687 (26286 protein atoms plus five epibatidine molecules, 27357 water molecules, 255 POPC lipids, 77 Na^+ and 83 Cl^- ions, corresponding to 100 mM solution, added to neutralize the new net system charge). The system was then equilibrated along a 0.5 μs simulation, with the same protocol as for the wild type system,[8] above described.

As concerns PMF calculations in the wild type, the initial configurations for milestoning have been prepared by placing the target ion inside the protein along the z axis at distinct positions (the centers of the Voronoi cells) separated by 2 Å starting from the intracellular mouth (corresponding to z=-19 Å in our system) up to the extracellular mouth (corresponding to z=+93 Å), see blue and red dots in Figure S1.

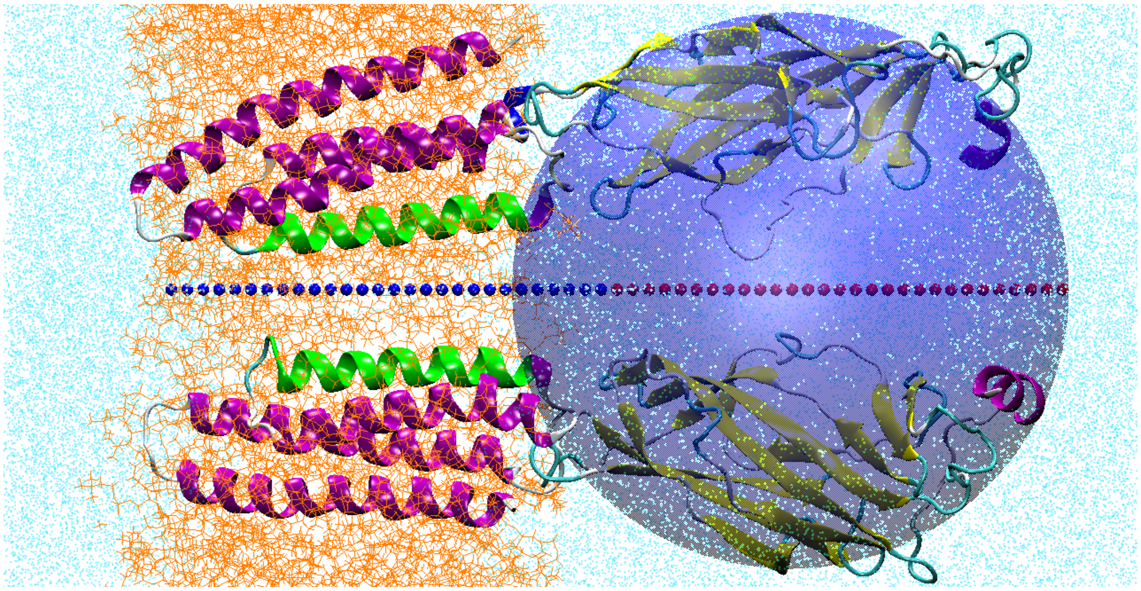

Figure S1: Section of the system simulated (wild type case): two subunits only are shown, represented in cartoon, for sake of clarity. Dots indicate the centers of the Voronoi cells. Blue dots, cells 1-28 (from -19Å to +35Å); red dots, cells 29 to 57 (from +37Å to +93Å). The "sphere" adopted to ensure the single ion condition in the LBD is showed in blue. Lipids are in yellow (in lines representation); water in cyan (represented with points).

As for the E-1′A mutant, a representative structure was taken from the last snapshot after 0.5 μs MD simulation; the initial system configurations for milestoning calculations were prepared by placing the target ion along the z axis at distinct positions (the centers of Voronoi cells) separated by 2 Å from the intracellular mouth (corresponding to z = -19 Å) up to the TMD/LBD interface (corresponding to$z$ = +35 Å), see blue dots in Figure S1.

Estimate of the single-channel conductance were based on the single-ion PMF according to Roux et al,[11] by exploiting the PFM values in the TMD both in wild type and mutant. To this aim, the diffusion coefficients of ions inside the TMD pore were set to half of the experimental bulk value (i.e 1.33 nm^2/ns for sodium and 2.03 nm^2/ns for chloride), as done

in Ref.[12] for simulations of *Torpedo* nAChrs.

Trajectory analysis

Stability assessment

To assess the overall stability of the E-1′A mutant channel, we calculated: i) the Cα atoms Root Mean Square Deviation (RMSD) from the initial configuration, along the 0.5μs trajectory, separately for the LBD and the TMD of the five subunits (in the following called P1,. . . , P5) and for the whole TMD and LBD; ii) the Cα atoms Root Mean Square Fluctuations (RMSFs) with respect to the average positions calculated over the last 200 ns segment of the equilibrium 0.5 μs trajectory. Results are shown in Figure S2 and Figure S3.

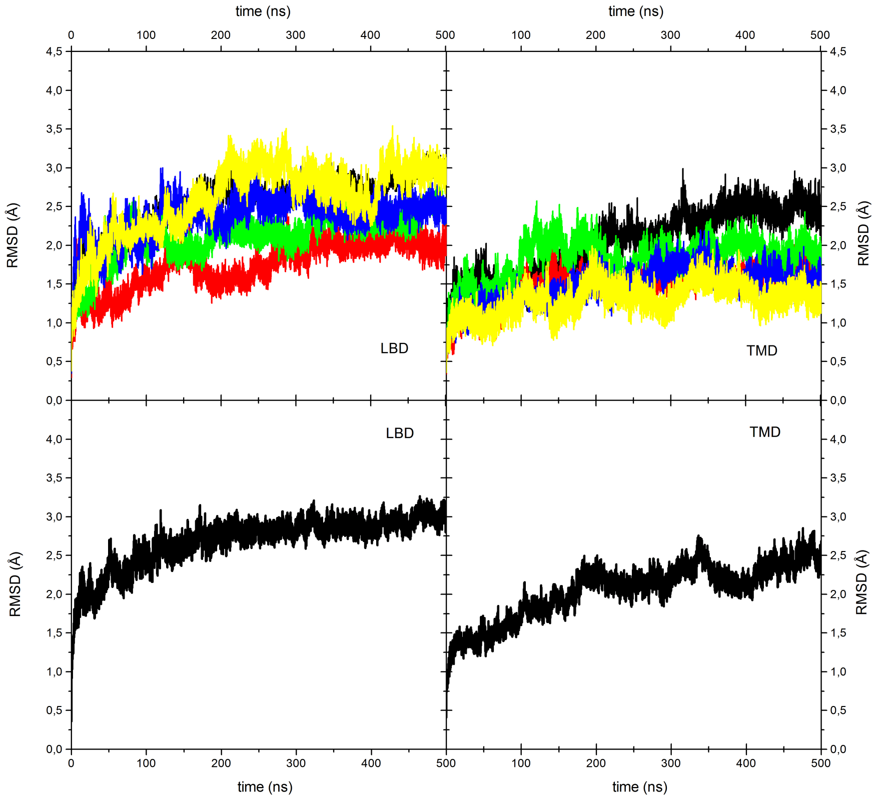

Figure S2: Upper panels: Cα Root Mean Square Deviation of individual E-1′ α7 subunits calculated from the starting conformation. Black curve: subunit P1; red curve: subunit P2; green curve: subunit P3; blue curve subunit P4; yellow curve: subunit P5. Lower panels: Cα Root Mean Square Deviation of the E-1′ α7 LBD and TMD, calculated from the starting conformation.

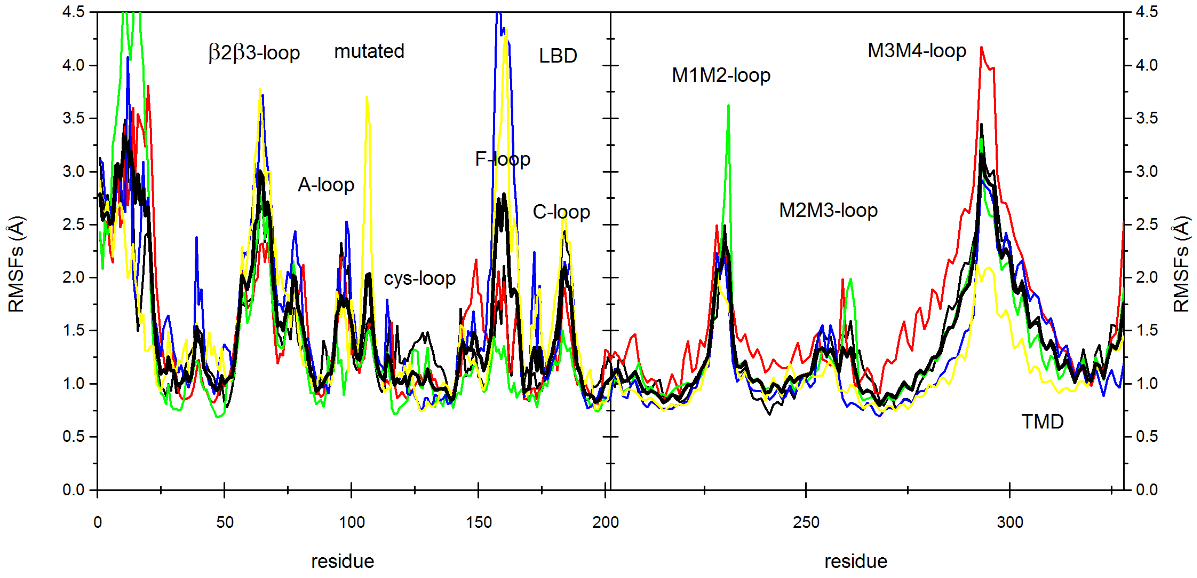

Figure S3: Cα RMSFs in the individual E-1′ α7 subunits with respect to the average structure calculated along the last 200 ns segment of the trajectory. The curves are colored as in Figure S1. The bold black line is the average over the five subunits. A-loop (residues 86–102), Cys-loop (121–135), F-loop (154–168), C-loop (179–188), M1-M2 loop (226–233), M3-M4 loop (285–304) are labelled.

Structure and hydration of the pore channel

Structure and hydration of the E-1′A channel pore were investigated as for the wild type protein[8,13,14] by calculating: i) the pore radius, computed using HOLE[15] on the structure averaged along the last 200 ns segment of the equilibrium trajectory; ii) polar and azimutal tilting of the M2 helices in TMD, defined as in ref.;[14] iii) the time evolution of the number of water molecules, along the same trajectory, in two regions: the full pore channel, as enclosed by the five M2 helices between G-2′ and E20′ (30 Å long) and in the hydrophobic region between L9′ and L16′ (10 Å long). Quaternary structural descriptors have been analyzed as well, as in ref.[14] Results are shown in Figure S4, Figure S5 and Figure S6.

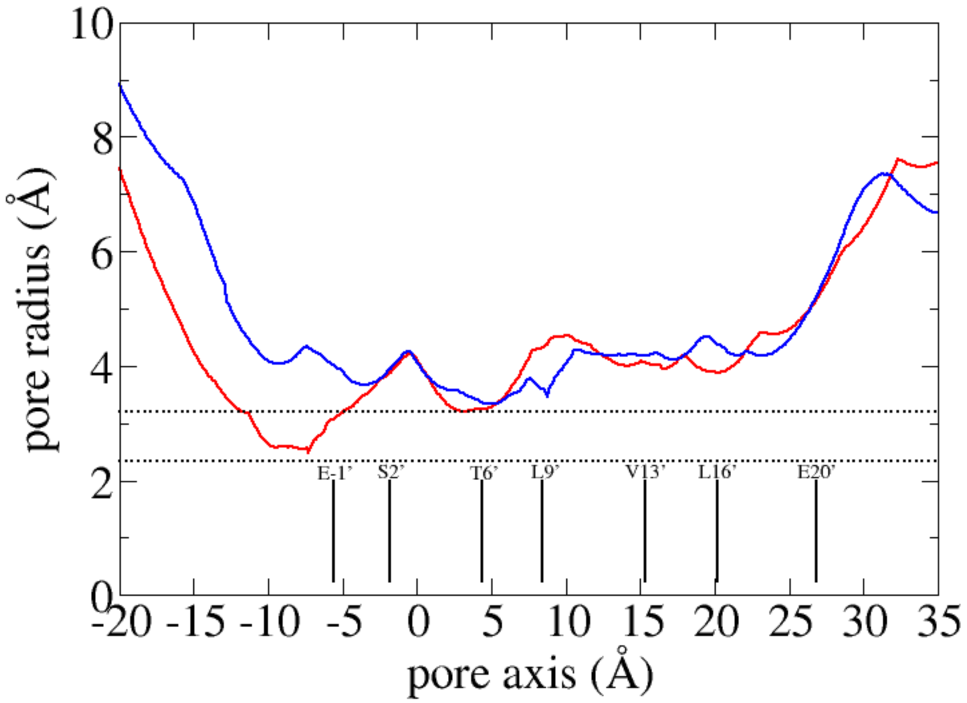

Figure S4: Pore radius profiles along the channel axis in the TMD of the modeled $\alpha7$, in the wild type (red curve)[8] and in the mutant (blue curve), computing using HOLE[15] on structures averaged over the last 200 ns of each equilibrium trajectory. Position of the M2 key residues are indicated. Radii of hydration shell for sodium (2.35 Å) and chloride (3.23 Å), respectively, are also indicated with dotted lines. The radii values are taken from ref.[16]

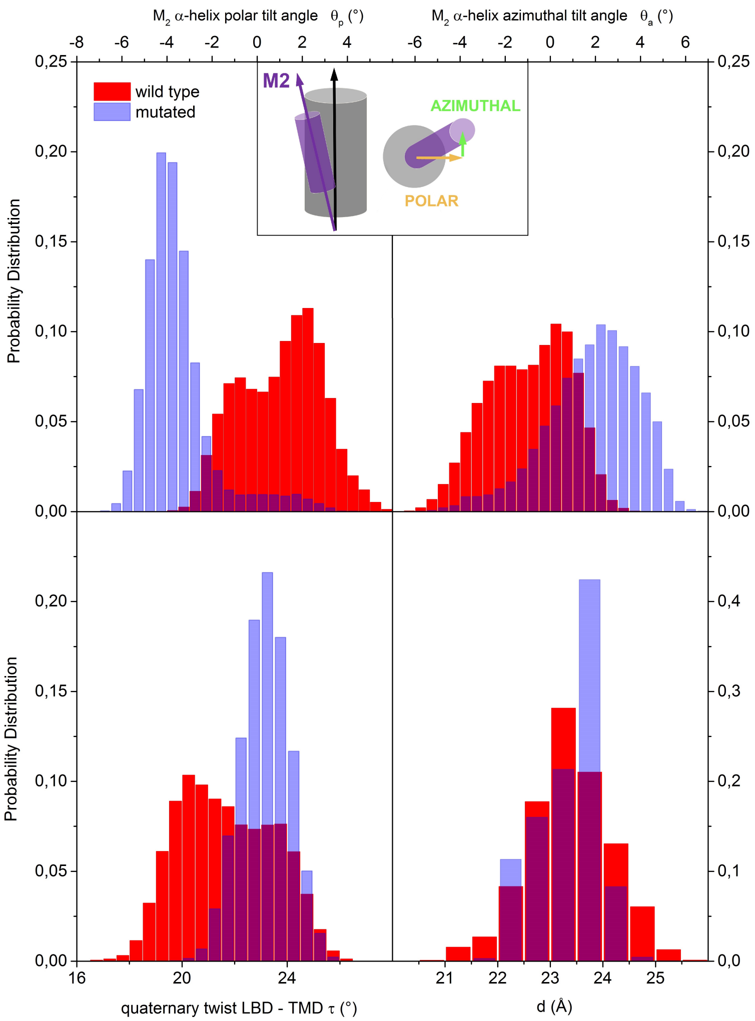

Figure S5: Upper left panel: distribution of the M2 polar tilt angle; upper right panel: distribution of the M2 azimuthal tilt angle. Data averaged over the five subunits. Lower left panel: distribution of LBD/TMD twist angle. Data averaged over the five subunits. Lower right panel: distribution of the LBD blooming distance (d). Data collected from the five subunits. Blue bars: E-1′A; red bars: wild type.

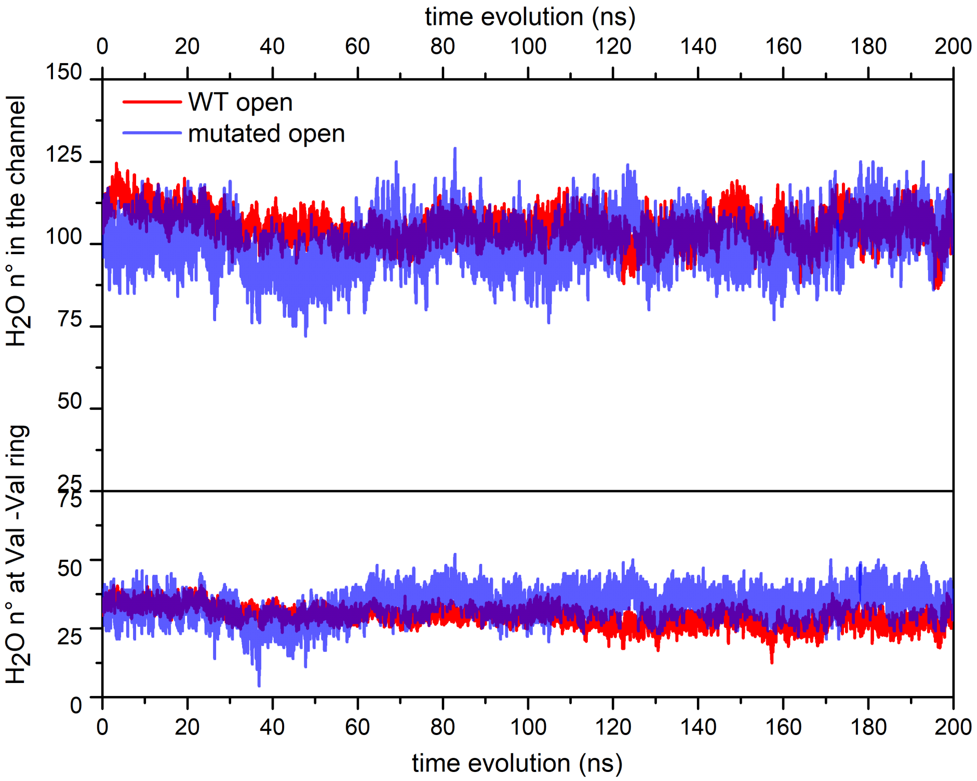

Figure S6: Upper panel: time evolution of water count in the pore lumen lined by the M2 helices. Lower panel: time evolution of water count in a region of 10 Å length, centered at V13′. Red curve: active;[14] blue curve: mutant (along the final 200 ns portion of the equilibrium simulation.)

Electrostatic potential map

Protein electrostatic potential maps have been calculated using the PMEpot plugin in VMD.[17] The electrostatic potential is calculated using all the protein atoms, a three dimensional grid of 100 × 100 × 144 points (which ensures at least one grid point *per* Å in each direction), and an Ewald factor of 0.25. The electrostatic potential generated by PMEpot is in units of kT/e; at T = 310 K, one PMEpot unit of electrostatic potential is equivalent to 27 mV. Results are shown in S7 and Figure S8.

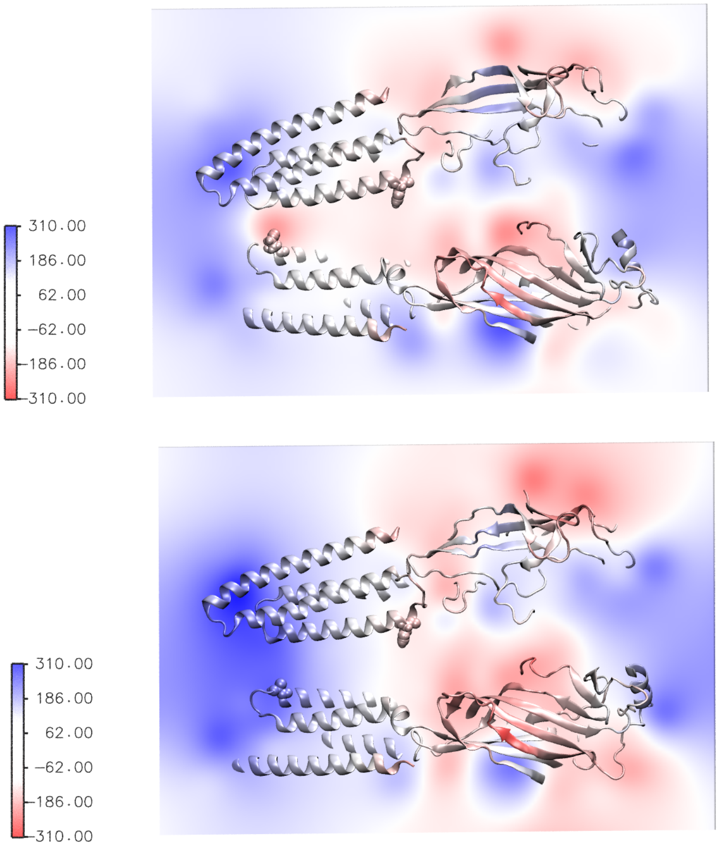

Figure S7: Protein electrostatic potential map, calculated on the structure averaged along a 200ns segment of the equilibrium trajectory of both wild type and E-1′A mutant. For sake of clarity, only two protein subunits (P3 and P5) are represented, in cartoon, colored according the electrostatic potential value. Upper panel: wild type; E-1′ (E888) and E20′ (E1565) are represented in vdw. Lower panel: E-1′A mutant. A-1′(A888) and E20′ (E1565) are represented in vdw. One PMEpot unit of electrostatic potential, at T = 310K, is equivalent to 27mV.[17]

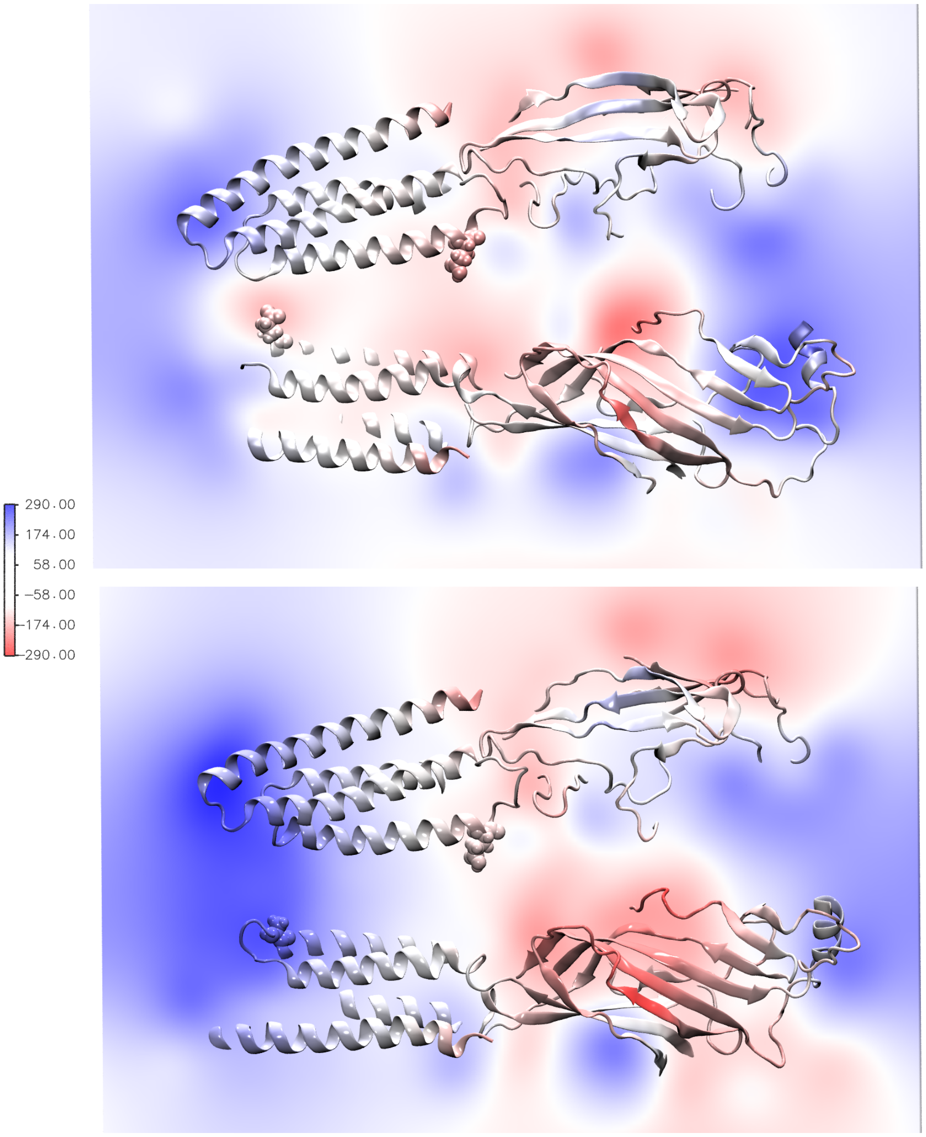

Figure S8: As in S6, map calculated by averaging along the chloride trajectory restricted in the Voronoi cell centered at the V13′ site.

E20′ dynamics in the E-1′A structure

Side-chain dynamics of the E20′ residues interacting with chloride has been investigated by monitoring the time evolution of the C-Cα-Cβ-Cγ torsion angle. Results are shown in Figure S9.

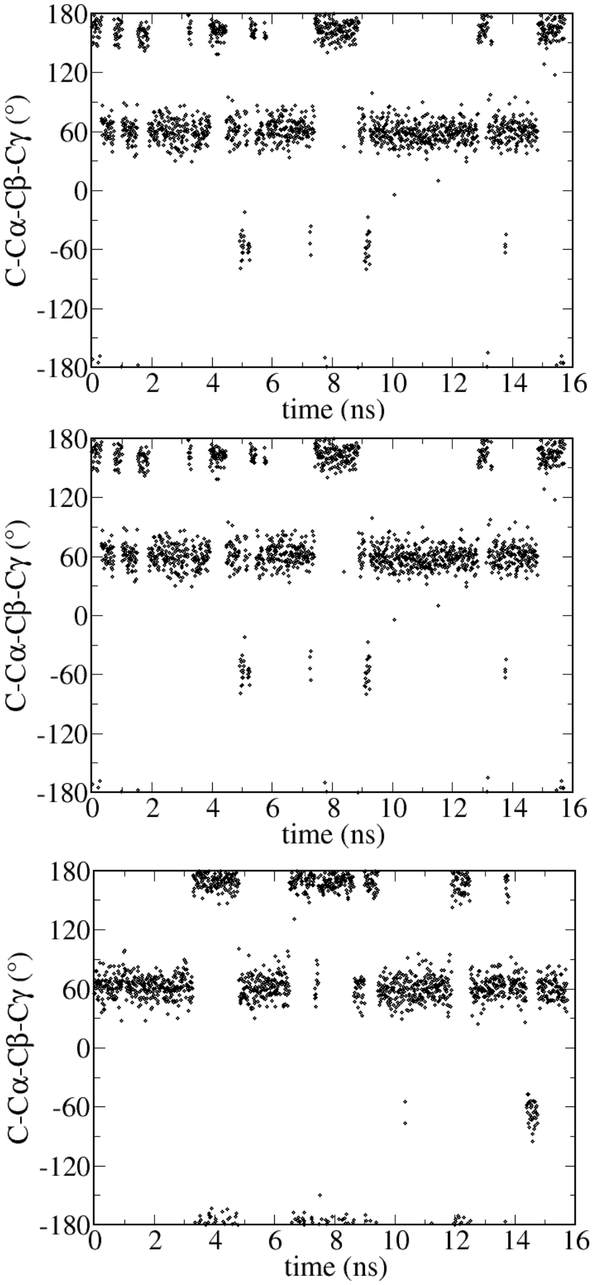

Figure S9: Time evolution of the C-Cα-Cβ-Cγ angle of three E20′ residues, along the trajectory restricted in the Voronoi cell at the V13′ site. From top to bottom: E253, E909 and E1237, belonging to subunit P1, P3 and P4, respectively.

References

(1) Quigley, D.; Probert, M. Langevin Dynamics in Constant Pressure Extended Systems. *J. Chem. Phys.* **2004**, *120*, 11432–11441.

(2) Phillips, J. C.; Braun, R.; Wang, W.; Gumbart, J.; Tajkhorshid, E.; Villa, E.; Chipot, C.; Skeel, R. D.; Kalé, L.; Schulten, K. Scalable Molecular Dynamics with NAMD. *J. Comput. Chem.* **2005**, *26*, 1781–1802.

(3) Wang, J.; Wang, W.; Kollmann, P.; Case, D. Antechamber, An Accessory Software Package For Molecular Mechanical Calculation. *J. Comput. Chem.* **2005**, *25*, 1157–1174.

(4) Frisch, M. J.; Trucks, G. W.; Schlegel, H. B.; Scuseria, G. E.; Robb, M. A.; Cheeseman, J. R.; et al., Gaussian 03, Revision B.02. *Pittsburgh PA: Gaussian, Inc.* **2003**,

(5) Feller, S. E.; Zhang, Y.; Pastor, R. W.; Brooks, B. R. Constant pressure molecular dynamics simulation: The Langevin piston method. *J. Chem. Phys.* **1995**, *103*, 4613.

(6) Essmann, U.; Perera, L.; Berkowitz, M.; Darden, T.; Lee, H.; Pedersen, L. G. A smooth particle mesh Ewald method. *J. Chem. Phys.* **1995**, *103*, 8577–8593.

(7) Ryckaert, J. P.; Ciccotti, G.; Berendsen, H. J. C. Numerical integration of the cartesian equations of motion of a system with constraints: molecular dynamics of n-alkanes. *J. Comput. Phys.* **1977**, *23*, 327–341.

(8) Chiodo, L.; Malliavin, T. E.; Maragliano, L.; Cottone, G.; Ciccotti, G. A Structural Model of the Human α7 Nicotinic Receptor in an Open Conformation. *PloS ONE* **2015**, *10*, e0133011.

(9) Bahsford, D.; Karplus, M. pKa of Ionizable Groups in Proteins: Atomic Detail from a Continuum Electrostatic Model. *Biochemistry.* **1990**, *29*, 10219–10225.

(10) Anandakrishnan, R.; Aguilar, B.; Onufriev, A. H++ 3.0: Automating pK Prediction and the Preparation of Biomolecular Structures for Atomistic Molecular Modeling and Simulation. *Nucleic Acids Res.* **2012**, *40*, 537–541.

(11) Roux, B.; Allen, T.; Bernèche, S.; W.Im, Theoretical and computational models of biological ion channels. *Quarterly Review of Biophysics* **2004**, *37*, 15–103.

(12) Beckstein, O.; M.P.Samson, A hydrophobic gate in an ion channel: the closed state of the nicotinic acetylcholine receptor. *Phys. Biol.* **2006**, *3*, 147–159.

(13) Chiodo, L.; Malliavin, T. E.; Maragliano, L.; Cottone, G. A Possible Desensitized State Conformation of the Human α7 Nicotinic Receptor: A Molecular Dynamics Study. *Biophys. Chem.* **2017**, *229*, 99–109.

(14) Chiodo, L.; Malliavin, T. E.; Giuffrida, S.; Maragliano, L.; Cottone, G. Closed-Locked and Apo-Resting State Structures of the Human 7 Nicotinic Receptor: A Computational Study. *J. Chem. Inf. Model* **2018**, *11*, 2278–2293.

(15) Smart, O. S.; Neduvelil, J. G.; Wang, X.; Wallace, B. A.; Sansom, M. S. P. HOLE: A Program for the Analysis of the Pore Dimensions of Ion Channel Structural Models. *J. Mol. Graph.* **1996**, *14*, 354–360.

(16) Mancinelli, R.; Botti, A.; Bruni, F.; Ricci, M. A.; Soper, A. K. Hydration of sodium, potassium, and chloride ions in solution and the concept of structure maker/breaker. *J. Phys.Chem.B* **2007**, *111*, 13570–13577.

(17) Aksimentiev, A.; Schulten, K. Imaging alpha-hemolysin with molecular dynamics: Ionic conductance, osmotic permeability and the electrostatic potential map. *Biophys. J.* **2005**, *88*, 3475–3761.
